# Natural Product‐Derived Vesicles in Nanotherapeutics

**DOI:** 10.1002/jev2.70367

**Published:** 2026-09-11

**Authors:** Kartiko Arif Purnomo, Tsong‐Long Hwang

**Affiliations:** ^1^ Center for Drug Research and Development Chang Gung University of Science and Technology Taoyuan Taiwan; ^2^ Graduate Institute of Health Industry Technology, College of Human Ecology Chang Gung University of Science and Technology Taoyuan Taiwan; ^3^ Department of Anesthesiology Chang Gung Memorial Hospital Taoyuan Taiwan; ^4^ Department of Chemical Engineering Ming Chi University of Technology New Taipei City Taiwan; ^5^ Graduate Institute of Natural Products, College of Medicine Chang Gung University Taoyuan Taiwan

**Keywords:** extracellular vesicles, nanotherapeutics, nanovesicles, natural product‐derived vesicles, natural products, plant‐derived nanovesicles

## Abstract

Natural product‐derived vesicles (NPVs) are lipid bilayer‐enclosed nanovesicles isolated from diverse sources, including plants, fungi, and marine organisms, and are increasingly recognized as an emerging class of extracellular vesicles with potential therapeutic relevance. Their abundance, renewability, and capacity to transport endogenous metabolites and exogenous cargoes highlight their potential as scalable biological delivery vehicles. NPVs can interact with mammalian cells, participate in intercellular communication, and influence conserved biological pathways that modulate disease, support tissue repair, and maintain cellular homeostasis. In addition, their structural plasticity provides opportunities for physicochemical optimization to improve functional performance and targeting properties. However, the development and translation of NPVs are constrained by challenges, including heterogeneity, variability in isolation procedures, and insufficient physicochemical and functional characterization. This review presents a systematic framework encompassing source identification, purification strategies, and characterization methods, while integrating separation approaches with multifaceted assessments to facilitate the rational development of NPVs, with particular emphasis on recent advances in structural optimization and therapeutic efficacy. Furthermore, the status of nine clinical trials is highlighted, reflecting the growing translational and therapeutic potential of NPVs. By delineating current limitations and discussing potential solutions, this review provides practical guidance for improving the physicochemical consistency and biological performance of NPVs, thereby facilitating their development as nature‐inspired nanotherapeutic systems.

## Graphical abstract

Natural product‐derived vesicles (NPVs) are an emerging nature‐based nanotherapeutic that can deliver bioactive metabolites into the target site and induce beneficial mechanisms. A practical, chronological bench‐to‐bedside approach throughout the entire NPV development process is expected to resolve challenges encountered in NPV studies. A comprehensive strategy, encompassing source selection, combination of isolation methods, physicochemical characterization, biotherapeutic evaluation, and structural refinement by aligning with existing knowledge and regulations, is essential for developing NPVs as nature‐based nanotherapeutics.

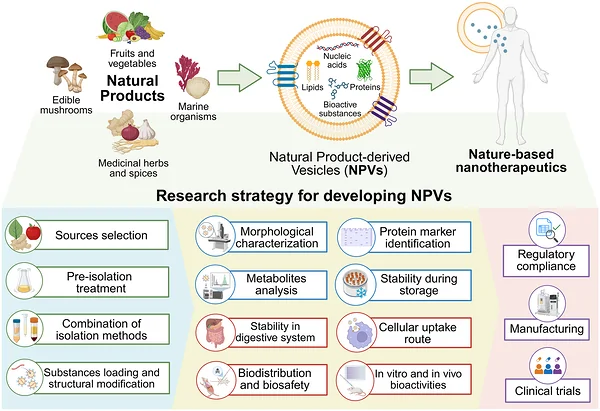

**Abbreviations**
1,2‐DLPC: 1,2‐dilauroyl‐sn‐glycero‐3‐phosphocholine
5‐HT: 5‐hydroxytryptamine
AEC: anion‐exchange chromatography
AFM: atomic force microscopy
ALT: alanine aminotransferase
ATPS: aqueous two‐phase system
BBB: blood‐brain barrier
BCA: bicinchoninic acid
BUN: blood urea nitrogen
C‐CP: capillary‐channeled polymer
COA: clinical outcome assessments
COI: concepts of interest
COX: cyclooxygenase
DLS: dynamic light scattering
DMSO: dimethyl sulfoxide
DSS: dextran sodium sulfate
ELD: electrophoretic dialysis
ELISA: enzyme‐linked immunosorbent assay
ELS: electrophoretic light scattering
EMA: european medicines agency
ESCRT: endosomal sorting complex required for transport
EVs: extracellular vesicles
EXPO: exocyst‐positive organelle
GLUT: glucose transporters
GMP: good manufacturing practice
GO: gene ontology
GPI: glycosylphosphatidylinositol
HFD/STZ: high‐fat diet/streptozotocin
HIC: hydrophobic interaction chromatography
HSP: heat shock proteins
HUVEC: human umbilical vein endothelial cells
IDO1: indoleamine 2,3‐dioxygenase 1
IFN‐γ: interferon‐gamma
IL: interleukin
IND: investigational new drug
iNOS: inducible nitric oxide synthase
ISEV: international society for extracellular vesicles
KEGG: kyoto encyclopedia of genes and genomes
LC‐MS/MS: liquid chromatography‐tandem mass spectrometry
LDL: low‐density lipoprotein
LPS: lipopolysaccharide
MFDS: ministry of food and drug safety
MISEV: minimal information for studies of extracellular vesicles
MVB: multivesicular bodies
MWCO: molecular weight cut‐off
MyD88: myeloid differentiation primary response 88
NanoFCM: nano flow cytometry
NCT: national clinical trial
NO: nitric oxide
NPVs: natural product‐derived vesicles
NTA: nanoparticle tracking analysis
QMS: quality management system
PDNVs: plant‐derived nanovesicles
PEG: polyethylene glycol
PMDA: pharmaceuticals and medical devices agency
ROS: reactive oxygen species
RT‐qPCR: reverse transcription quantitative polymerase chain reaction
SDS: sodium dodecyl sulfate
SEC: size‐exclusion chromatography
TEM: transmission electron microscopy
TFDA: taiwan food and drug administration
TMAO: trimethylamine N‐oxide
tMCAO: transient middle cerebral artery occlusion
TNF‐α: tumor necrosis factor‐alpha
TFF: tangential flow filtration
TLR4: toll‐like receptor 4
US FDA: US food and drug administration
VEGF: vascular endothelial growth factor

## 1 Introduction

Natural products are valuable sources of bioactive compounds and remain widely used in healthcare worldwide. Owing to their inherent compatibility with human biological systems, natural product sources generate bioactive molecules with favourable biosafety profiles and the capacity to modulate specific molecular and cellular mechanisms (Liu et al. 2025; Lv et al. 2024; Mehta et al. 2015). Despite their favourable biosafety profiles and therapeutic potential, poor solubility, stability, membrane permeability, and absorption often restrict their bioavailability and clinical efficacy (Liu et al. 2025; Lv et al. 2024).

Extracellular vesicles (EVs) are lipid bilayer‐enclosed nanoscale particles naturally released by cells that mediate intercellular communication through the transfer of proteins, lipids, nucleic acids, and metabolites (Hao et al. 2024; Lian et al. 2022; Mao et al. 2024). Their high biocompatibility, low toxicity, and cargo‐protective properties have attracted considerable interest for therapeutic delivery and disease diagnosis (Jeppesen et al. 2023; Moholkar et al. 2023; Tenchov et al. 2022). EV biogenesis occurs through several pathways, primarily via the multivesicular body (MVB) pathway, while alternative mechanisms such as vacuolar secretion during pathogen defense and exocyst‐positive organelle (EXPO)‐mediated secretion also contribute to EV formation under specific biological conditions (Gandham et al. 2020; Sha et al. 2024; Zhao, Lin, et al. 2024). Despite these advantages, EVs derived from cultured human cells and biofluids typically yield low amounts, posing a major challenge for large‐scale manufacturing and clinical translation. The generation of therapeutically relevant EV doses often requires substantial volumes of conditioned media and repeated cell expansion, resulting in high costs, scalability limitations, and ethical concerns (Giancaterino and Boi 2023; Haraszti et al. 2018).

**FIGURE 1:**
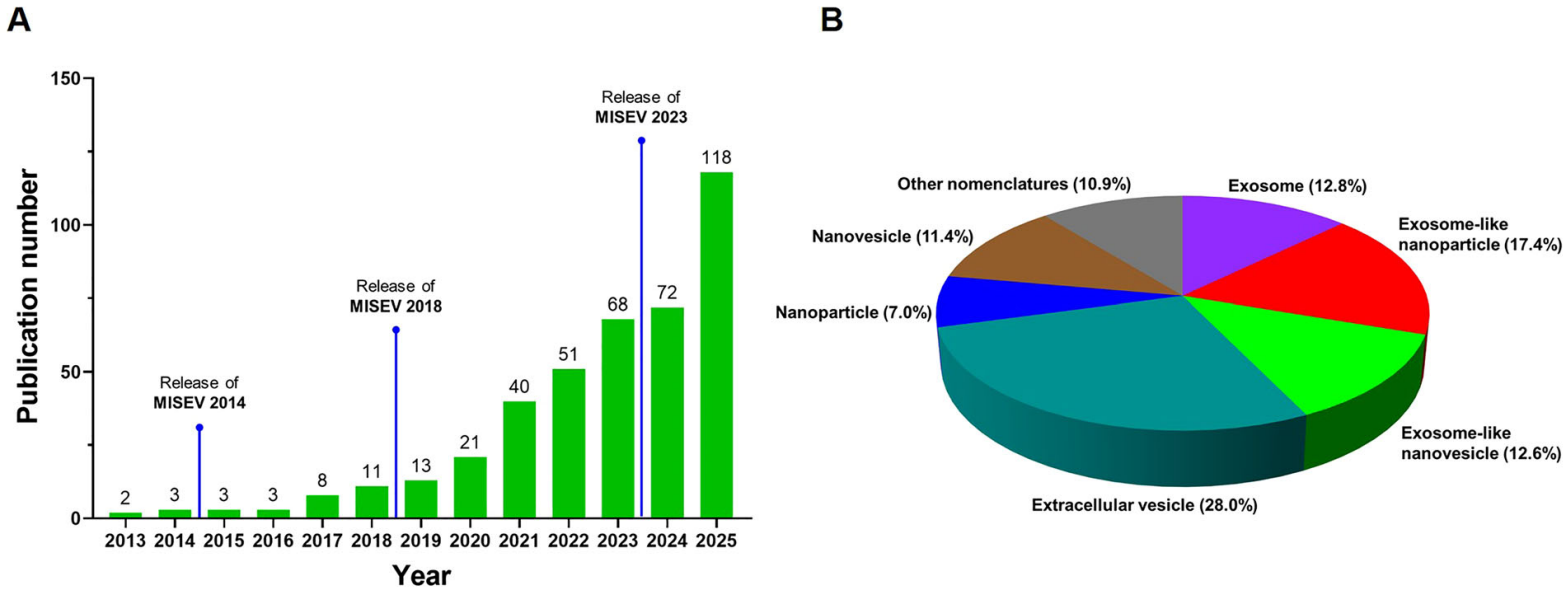
(A) Number of published research articles about NPVs and (B) NPV nomenclatures in research articles from 2013 to 2025. Data were collected via a literature search in databases, with the criteria of including only original research articles and nanovesicles isolated from natural product sources, published between 2013 and 2025.

Natural product‐derived vesicles (NPVs) originate from diverse natural sources, including herbs, spices, fruits, vegetables, grains, edible fungi, and marine organisms, and naturally encapsulate bioactive metabolites from their parental sources (Giancaterino and Boi 2023; Lian et al. 2022; Zhao, Lin, et al. 2024). The term NPVs encompasses both natural EVs and other naturally‐derived nanovesicles, including plant‐derived nanovesicles (PDNVs). Natural EVs describe the isolated NPVs from apoplastic fluids, conditioned media of suspension cultures, secreted plant exudates, and phloem and xylem saps using non‐destructive pre‐isolation treatment. PDNVs represent purified NPVs from plant tissues via destructive processing and therefore cannot confirm the natural release of EVs into the extracellular space (Pinedo et al. 2021). Their abundant availability, scalability, relatively low production costs, and the edible nature of many source materials make NPVs attractive candidates for large‐scale manufacturing and clinical translation, with favourable biocompatibility and safety profiles (Fang and Liu 2022; Giancaterino and Boi 2023; Ly et al. 2023; Paganini et al. 2019). NPVs represent a relatively new research field with unresolved challenges in isolation techniques, large‐scale production, clinical translation, and regulatory compliance. Although EVs released from the mesophyll cells of *Triticum dicoccum* and the meristematic cells of carrot were reported in the 1960s, their biological significance remained largely unexplored and was subsequently overshadowed by the rapid development of mammalian EV research (Couch et al. 2021; Halperin and Jensen 1967; Ly et al. 2023; Manocha and Shaw 1964). Interest in plant‐derived EVs was renewed in 2009 when vesicles isolated from sunflower extracellular fluids were reported to mediate intercellular communication in plants (Regente et al. 2009). Subsequent studies demonstrated the therapeutic potential of NPVs, including grape PDNVs that attenuated colitis and nanovectors from grapefruit PDNV that inhibited brain tumor growth (Ju et al. 2013; Wang et al. 2013). Since then, NPV research has expanded rapidly to elucidate their biological characteristics and therapeutic functions, particularly following the publication of the Minimal Information for Studies of Extracellular Vesicles (MISEV) 2018 guidelines (Figure 1A).

Although the International Society for Extracellular Vesicles (ISEV) released MISEV 2023 as its latest guidance, specific standardized recommendations for NPVs remain unavailable (Pinedo et al. 2021). The general term ‘extracellular vesicle’ is encouraged to avoid assumptions regarding EV characteristics and biogenesis based on the cell of origin (Thery et al. 2018; Welsh et al. 2024). However, only 28.0% of NPV studies adopted this terminology, whereas inadvisable terms such as ‘exosome’ (12.8%), ‘exosome‐like nanovesicle’ (12.6%), and ‘exosome‐like nanoparticle’ (17.4%) remain frequently used (Figure 1B). The use of the term ‘exosome’ is discouraged unless the biogenesis pathway can be conclusively demonstrated through direct evidence of EV release and specific markers of subcellular origin (Thery et al. 2018). Likewise, the classification of small (<200 nm) and large (>200 nm) EVs should be interpreted cautiously because definitive size boundaries have not been established (Welsh et al. 2024).

Beyond nomenclature issues, significant challenges persist in isolation, characterization, quality control, biosafety evaluation, scalable manufacturing, and clinical translation (Nguyen et al. 2020; Pinedo et al. 2021). To address these gaps, this review presents a systematic bench‐to‐bedside framework encompassing source identification, separation strategies, characterization methods, stability maintenance, biodistribution and biosafety assessment, vesicle engineering, large‐scale manufacturing, and regulatory considerations. By integrating recent advances with translational perspectives, this review aims to facilitate the rational development of NPVs as a robust nanotherapeutic platform.

## 2 Selection of NPV Sources

Selecting NPV sources is part of the quality‐by‐design framework, ensuring product quality from the outset of the development process (Khan and Smillie 2012). Natural product sources possess well‐documented capacities to modulate disease processes through specific mechanisms and bioactive constituents. The intrinsic therapeutic activities and toxicology of extracts or components from these sources should be carefully considered. Ethnopharmacological use, prior bioassays, and clinical evidence provide valuable insight into the anticipated bioactivity and potential applications of NPVs. Moreover, source selection should also account for practical and safety‐related factors, including availability, allergenic potential, cost, drug interactions, particle concentration, and cellular uptake efficiency. Sources capable of producing high yields of NPVs with efficient cellular uptake should be prioritized for NPV development (Corvigno et al. 2024). The accurate selection of NPV sources provides an excellent research foundation and helps avoid unwanted issues related to low yields, ineffective therapeutic effects, and high production costs.

NPV sources can be modified through pathogen infection and nutritional biofortification to enhance the levels of specific beneficial components in the vesicles. This process changes the metabolite content within vesicles but rarely affects vesicle morphology or membrane surface structure. Specific pathogen infection in the host can boost innate immunity for defense responses (Rutter and Innes 2017). *Aphys gossypii‐*infested melon plants secreted phloem sap EVs with higher expression of specific defense MG132 proteasome complex than non‐infested EVs (Sánchez‐López et al. 2024). Enhanced EV release in infested plants confirms the defense mechanism response of EVs by escalating fusion between MVB and the plasma membrane to increase EV formation during pathogen infection (Rutter and Innes 2017). Selenium‐biofortified broccoli PDNVs contained 25‐fold higher selenium content compared to normal broccoli PDNVs and exhibited significantly stronger anti‐pancreatic adenocarcinoma potency (Wang et al. 2023).

## 3 Optimization of NPV Isolation Methods

The strategy for enhancing NPV quality and quantity consists of pre‐isolation treatments and isolation methods. Performing pre‐isolation treatment is essential to provide initial material for the isolation step. A suitable isolation technique is essential for maximizing yield and improving purity.

### 3.1 Pre‐Isolation Treatment

Pre‐isolation treatment of natural product sources is a crucial step in obtaining material for NPV isolation and ensuring high yield and purity. Each treatment has its own advantages and disadvantages, typically categorized as destructive or less destructive (Table 1). Juice extraction via homogenization step, such as blending, grinding, or squeezing, is considered a destructive method that damages the plasma membrane and produces cellular fragments under pressure, thereby increasing the risk of NPV contamination by intracellular components. However, its simplicity and ability to provide substantial initial material make it a popular choice for pre‐treatment (Zhao, Lin, et al. 2024). Its modification by incubating *Cordyceps militaris* fruiting bodies and *Opuntia ficus‐indica* fruits in an isolation buffer before homogenization could lead to the accumulation of released PDNVs (Luo et al. 2024; Valentino et al. 2024).

**TABLE 1:** Principles, advantages, and disadvantages of pre‐isolation treatments for the NPV sources.

Treatments	Principles	Advantages	Disadvantages	Ref.
Homogenization	Applying pressure forces by blending, grinding, or squeezing to break tissue for extracting juice from the material source.	Convenient and quick procedures. Yielding a high amount of initial material.	Inducing cell damage due to broken plasma membrane and cellular fragments caused by pressure stress. Tissue rupture leads to NPV contamination by cytoplasm, nucleus, and intracellular components.	Zhao et al. (2024)
Tissue infiltration	Infiltrating vesicle isolation buffer into leaves or seeds to obtain apoplastic fluid in the extracellular spaces of cell membranes.	Minimum cell damage and tissue rupture to reduce NPV contamination. Avoiding destruction of material sources to use for metabolite analysis.	Low amount of initial material. Possible low NPV yield. Limited to small‐scale applications.	Wu et al. (2024)
Enzyme digestion	Adding digestive enzymes to degrade the cell wall, allowing NPV release into the surrounding media.	Applicable for destructive and less‐destructive methods.	Possible cell wall leaking if intensive degradation occurred.	Zhao et al. (2023)
Callus inoculation	Inoculating the callus in the controlled medium to grow it into a compact planar mass.	Sterile and controlled culture. Enhancing cell‐to‐cell communication to increase NPV yield.	Requiring a long time to generate callus. Limited to small‐scale applications. Potential co‐isolated protein.	Kocholata et al. (2024)
Hydroponic system	Applying a hydroponic system to living plants to collect a water solution containing root exudates.	Enabling additional nutrition supply in the hydroponic water.	Requiring a long time to allow root exudates to release into the water. Contamination risks from the hydroponic environment.	De Palma et al. (2020)

Tissue infiltration using a buffer has been developed as a gentler method to prevent cell damage and EV contamination. It has become a standard method for extracting apoplastic fluids from *Arabidopsis thaliana* leaves. Extracellular fluid extraction from sunflower seeds, achieved by applying half the standard infiltration pressure with Tris‐HCl buffer, resulted in lower electrolyte leakage due to reduced cell lysis (Regente et al. 2017). Although this method helps minimize NPV contamination by reducing cell damage, it is less preferred because of the small quantities of apoplastic fluid obtained for initial NPV isolation material (Wu et al. 2024). Soaking *Morinda officinalis* roots in vesicle isolation buffer with cellulase, pectinase, and hemicellulose, followed by gentle shaking without any physical destructive treatment, produced nearly three times of yield and a significant increase in protein concentration compared to the homogenization method. Plant cell walls are broken down by digestive enzymes to help NPVs be released into the surrounding cell wall environment (Zhao et al. 2023).

*Nicotiana tabacum* seed callus culture produced a higher EV yield than suspension culture and apoplastic fluid. Callus culture formed a close‐contact and compact mass in the culture medium, facilitating frequent cell‐to‐cell communication and thereby enhancing NPV production (Kocholata et al. 2024). The hydroponic system incubates roots in ultrapure water for an extended period to facilitate EV release without causing tissue damage. Tomato root EVs inhibited spore germination in plant‐pathogenic fungi (De Palma et al. 2020). Therefore, selective pre‐isolation treatment that employs a less‐destructive approach can potentially improve the yield and purity of isolated NPVs by increasing the initial isolation material and avoiding contamination.

### 3.2 Combination of Isolation Methods

**FIGURE 2:**
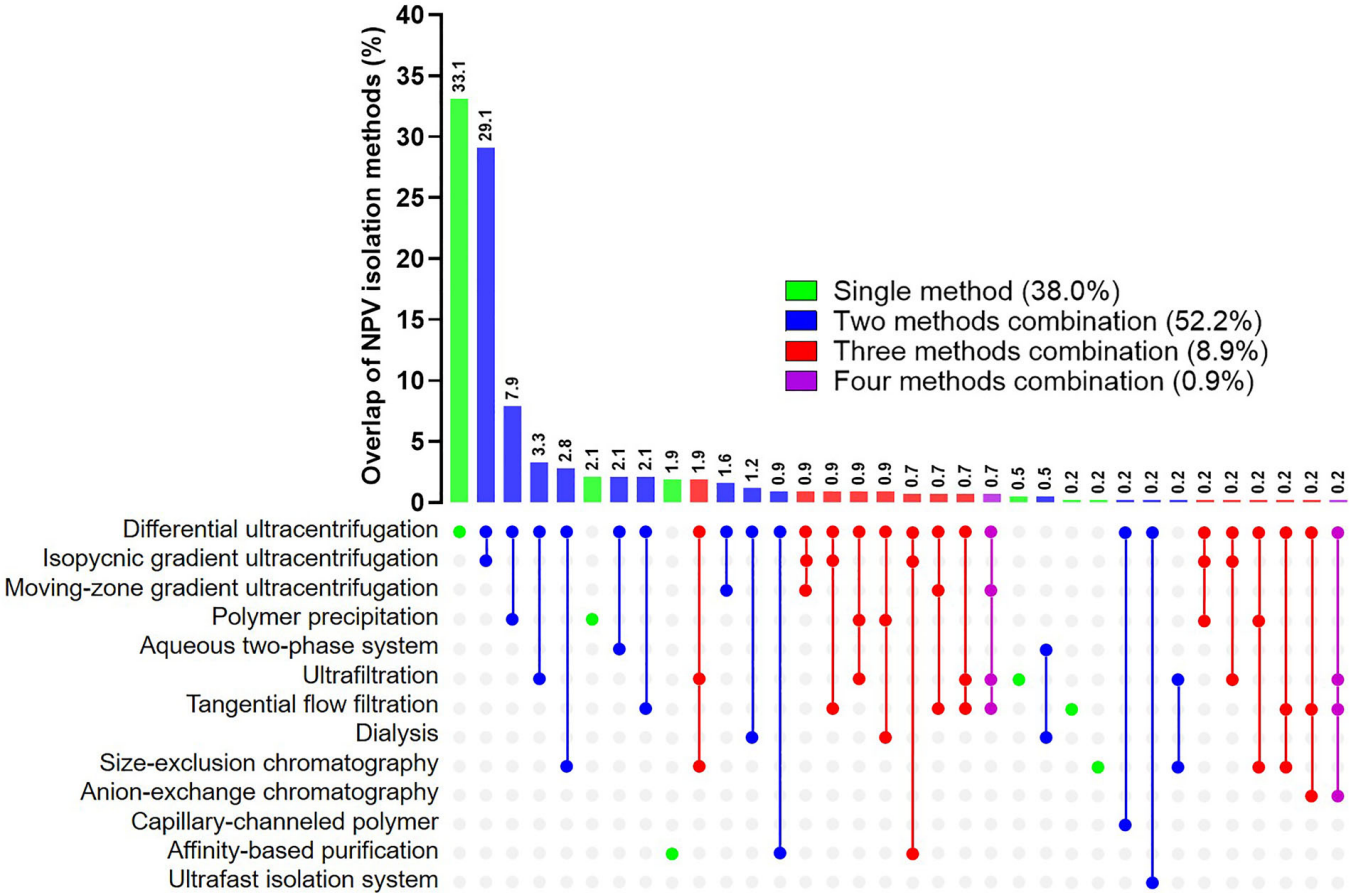
Overlap of single and multiple methods used for NPV isolation. Data were collected from 413 published original research articles from 2013 to 2025, illustrating the NPV isolation using a single method (green), a combination of two methods (blue), three methods (red), and four methods (purple).

Each isolation method has its own working principles and requires specific equipment and protocols, with distinct advantages and disadvantages that should be carefully considered (Table 2) (Giancaterino and Boi 2023; Lian et al. 2022; Yang, Zhang, et al. 2020). Although differential ultracentrifugation is commonly used as a standalone method, combining isolation techniques is preferred (Figure 2). A combination of multiple isolation techniques offers a highly selective and scalable method to obtain uniform NPVs with high yield and purity (Gandham et al. 2020; Liangsupree et al. 2021).

**TABLE 2:** Principles, advantages, and disadvantages of methods for isolating NPVs.

Methods	Principles	Advantages	Disadvantages	Ref.
Differential ultracentrifugation	Separating NPVs from impurities according to sedimentation rate differences by applying multiple gradual centrifugal forces.	Gold standard and common protocol for NPV isolation. No additional reagent to avoid contamination risk.	Potential vesicle damage and NPV aggregation. Limited yield and scalability. Low NPV purity.	Du et al. (2023); Praveena et al. (2025); Wang et al. (2024)
Isopycnic gradient ultracentrifugation	Adding medium layers of decreasing density gradient solutions from bottom to top before ultracentrifugation to separate NPVs from impurities with different buoyant densities.	High NPV purity. Enabling NPV subpopulations separation. Less NPV damage and aggregation.	Low scalability. Time‐consuming and labour‐intensive. Unable to distinguish impurities with a similar density to NPVs. Requiring a clean‐up step to remove the gradient reagent.	Du et al. (2023); Wang et al. (2024); Yang et al. (2020))
Moving‐zone gradient ultracentrifugation	Adding a high‐density cushion layer to the bottom of the lower‐density NPV‐containing medium before ultracentrifugation enables separation of NPVs based on mass, size, and density.	High purity. Less NPV damage and aggregation.	Prolonged centrifugation time can precipitate NPV and impurities into the cushion. Low scalability. Time‐consuming and labour‐intensive.	(Wang et al. 2024; Yang et al. 2020)
Polymer precipitation	Inducing NPV precipitation by adding a hydrophilic polymer to lower NPV solubility.	Requiring low‐speed centrifugation. Suitable for large sample volumes. Potential high yield. Relatively low cost.	Potential low purity due to co‐precipitation of NPVs and polymer. Requiring an additional clean‐up step.	Du et al. (2023); Wang et al. (2024); Yang et al. (2020)
Aqueous two‐phase system	Adding immiscible hydrophobic and hydrophilic solutions, followed by centrifugation to separate NPVs from contaminants based on different partition coefficients.	Requiring low‐speed centrifugation. Rapid procedure. Less NPV damage and aggregation.	Potential artifacts increase. Requiring repeated washing steps to remove PEG and dextran from NPV‐rich solution. Dextran may increase NPV viscosity.	Hatti‐Kaul (2001); Su et al. (2025); Yang et al. (2020)
Ultrafiltration	Using a specific pore size cut‐off membrane for applying ultracentrifugation to separate NPVs from impurities with different molecular sizes.	Obtaining NPVs with uniform size. Reducing NPV co‐isolation with contaminants.	Membrane fouling reduces NPV yield. High pressure may damage the NPV membrane. Small‐sized NPVs are lost because passing the membrane.	Fang and Liu (2022); Wang et al. (2024); Yang et al. (2020)
Tangential flow filtration	Applying a parallel flow of the feed stream on the membrane surface and recirculating the feed to repeat the filtration process for yielding a high‐concentrated NPV solution.	High NPV yield by avoiding membrane fouling. Suitable for large quantity samples and scaling‐up.	Requiring a set of specialized equipment and a specific filter type.	Paganini et al. (2019); Wang et al. (2024); Yang et al. (2020)
Dialysis	Separating NPVs from impurities by incubating in a membrane bag with a specific molecular size cut‐off between different concentration media.	Does not require additional reagents. Applying electric current may reduce purification time and improve NPV yield according to the electrical charge.	Possible membrane fouling. Heterogeneous NPV size and charge may reduce efficiency. Time‐consuming process.	Yang et al. (2020); Zheng et al. (2025)
Size‐exclusion chromatography	Passing NPV‐containing solution to porous stationary phase for separating NPVs from impurities with different retention rates based on hydrodynamic molecular size.	Minimizing co‐isolation of NPVs with impurities. Enabling NPV subpopulations purification. Preserving NPV structure by avoiding physical damage. Possible for large‐scale processing.	Requiring an additional step for concentrating NPVs. Time‐consuming. Unable to separate impurities with a similar size to NPVs; such as lipoprotein.	Dilsiz (2024); Paganini et al. (2019); Yang et al. (2020)
Anion‐exchange chromatography	Loading NPV‐containing solution into the column of ion‐exchange resin to separate NPVs from impurities via interaction between negatively‐charged NPV membrane and positively‐charged stationary phase.	High NPV purity by removing positively‐charged contaminants. Preserving NPV structure by avoiding physical damage.	Time‐consuming and complex process. Reduced NPV yield due to undesired NPV interaction with column matrix.	Seo et al. (2022); Silva et al. (2023); Wang et al. (2024)
Capillary‐channeled polymer	Using a capillary‐channeled polymer fibre as a stationary phase to facilitate high‐permeability fluid flow based on hydrophobic interaction by allowing a high‐to‐low salt transition to capture NPVs.	High NPV yield and purity. Quick and cost‐effective NPV isolation procedure. Less NPV damage risk.	Limited small sample capacity. Difficult for scaling up.	Huang et al. (2019); Jackson et al. (2023)
Affinity‐based purification	Adding an antibody‐specific NPV binding marker to capture NPVs from the solution medium based on antigen‐antibody interaction between NPV surface proteins and exogenous antibody.	High NPV purity due to high binding selectivity. Enabling specific NPV subpopulations isolation. Enabling the capture of NPVs from specific origins.	Requiring rigorous optimization for antibody selection. Low processing volume and sample capacity. Unable to detect and capture NPVs with different surface proteins. Difficult to remove binding antibody after isolation.	Dilsiz (2024); Du et al. (2023); Wang et al. (2024)
Microfluidics	Using a microchannel device to control fluid flow to capture NPVs based on affinity or size.	High NPV purity. Low sample volume. Quick processing time. Improving drug loading efficiency.	Requiring complex integrated equipment. Limited sample processing volume.	Dilsiz (2024); Kumar et al. (2023); Praveena et al. (2025)
Ultrafast isolation system	Applying dual transverse waves to vibrate the nanoporous filter membrane via an acoustofluidic stream.	Less membrane fouling. High yield and purity. Quick processing time. Excellent robustness and reproducibility.	Limited scalability and specificity. Unable to isolate NPV subpopulations with a narrow interval.	Chen et al. (2021)

Differential ultracentrifugation requires a relatively simple protocol and is considered the gold standard for general EV isolation (Yang et al. 2020). It offers balance between efficiency and purity by relying on the different precipitation rates based on particle size, since smaller particles, including EVs, need greater force and longer period of centrifugation than larger ones (Wang, Zhou, et al. 2024). Sequentially, low centrifugal forces (<10,000 × g) remove dead cells and debris, moderate forces (10,000‒20,000 × g) eliminate large vesicles, and high forces (100,000‒200,000 × g) precipitate EVs pellet (Du et al. 2023). However, it requires a long processing time, potentially induces EV damage after colliding with a solid‐bottom vessel at high centrifugal speed, and reduces EV purity due to contaminant aggregation (Du et al. 2023; Praveena et al. 2025; Wang, Zhou, et al. 2024). Therefore, coupling differential ultracentrifugation with subsequent purification techniques, such as density gradient ultracentrifugation, dialysis, ultrafiltration, and chromatography, is necessary to diminish its limitations (Wang, Zhou, et al. 2024; Webber and Clayton 2013).

The most common paired method for NPV isolation is differential ultracentrifugation with an isopycnic gradient (Figure 2). Pre‐concentrated EVs are loaded onto multi‐density gradient solution layers, then ultracentrifuged to separate EV subpopulations from contaminants by density (Du et al. 2023; Wang, Zhou, et al. 2024). Although sucrose is more frequently used for a gradient solution layer, iodixanol (OptiPrep) offers greater stability, lower viscosity, and better preservation of vesicle integrity (Dilsiz 2024). Despite a reduced total particle concentration, adding a sucrose density gradient purification step after ultracentrifugation significantly increased the proportion of ginger PDNVs. Moreover, it had significantly lower zeta potential than isolated ginger PDNVs via membrane filtration and polymer precipitation, indicating that density gradient ultracentrifugation can remove a substantial portion of charged contaminants (Ming et al. 2025). It also better maintained miRNA content in *Pinctada fucata* oyster nanovesicles than ultracentrifugation (Meng et al. 2025). Iodixanol density gradient ultracentrifugation enabled the purification of *A. thaliana* apoplastic wash fluid EVs within the density range of 1.04‒1.12 g/mL (Koch et al. 2025). Pairing tangential flow filtration (TFF) and ultracentrifugation with OptiPrep gradient purification enhanced particle number and purity of green tea‐derived *Lactobacillus plantarum* EVs (Kim, Lee, et al. 2020). The limited interface space between density zones may reduce EV yield, while a prolonged ultracentrifugation step can alter vesicular structure (Wang, Zhou, et al. 2024; Yang, Zhang, et al. 2020).

Moving‐zone gradient ultracentrifugation consists of a low‐density layer on top and a high‐density cushion layer at the bottom. EVs are purified above the cushion layer because they have a higher density than the solution in the top layer, whereas insoluble contaminants can be pelleted at the bottom of the vessel (Yang, Zhang, et al. 2020). Layering ginseng PDNV‐containing solution on top of 27% and 68% sucrose layers, followed by high‐speed centrifugation, led to ginseng PDNV partition at the interface layer, serving as an effective pre‐treatment (Kim et al. 2023; Li et al. 2023). Excess sucrose in the *Catharanthus roseus* PDNV layer was eliminated using ultrafiltration (Ou et al. 2023). The cushion reduces EV damage by preventing collision with the vessel base. However, prolonged centrifugation can result in EVs co‐pelleting with other material contaminants (Wang, Zhou, et al. 2024; Yang, Zhang, et al. 2020).

EV isolation using polymer precipitation is conducted by incubating EV‐containing media with polymer solution, followed by low‐speed centrifugation. This method offers simplicity and requires non‐sophisticated equipment, forming the basis of several commercial EV isolation kits. Polyethylene glycol (PEG) with a typical molecular weight of 6‒20 kDa is predominantly used as a precipitating agent due to its high hydrophilic nature and ability to alter solute solubility in water to promote EV precipitation (Du et al. 2023; Yang, Zhang, et al. 2020). The efficiency of this method is affected by polymer concentration and selected molecular weight, along with solution pH, temperature, and incubation duration (Wang, Zhou, et al. 2024). Despite having a larger particle size, PEG6000‐based precipitation of *A. thaliana* EVs showed no expression of PR1 marker and stronger expression of TET8 marker than using 10 kDa ultrafiltration, representing a better ability to discard apoplastic fluid contaminants (Liu et al. 2020). This method induces co‐precipitation of EVs with non‐vesicular and lipoprotein impurities, which can significantly reduce EV purity (Du et al. 2023; Wang, Zhou, et al. 2024). PEG precipitation gave significantly lower purity of cabbage PDNVs than size‐exclusion chromatography, indicating co‐isolation of non‐vesicular particulates (You et al. 2021). Therefore, pre‐treatment by centrifugation and post‐purification by dialysis or size‐exclusion chromatography are essential to minimize impurities (Yang, Zhang, et al. 2020).

Aqueous two‐phase system (ATPS) is a water‐based biphasic system using two immiscible polymers of PEG and dextran in aqueous medium. It is suitable for separating cell subpopulations and purifying proteins due to its low interphase tension and high‐water content (Hatti‐Kaul 2001; Su et al. 2025). Low‐speed centrifugation separates EV accumulation in the dextran phase from proteins and impurities in the PEG phase (Yang, Zhang, et al. 2020). Celery root PDNVs and grapefruit PDNVs were purified in the solution of PEG‐dextran with a ratio of 7.7:3.3. PEG phase removal using multiple washing steps eliminated cellular debris from EVs in the dextran phase (Savcı et al. 2021; Tasli 2022). Despite its promising features as an inexpensive and rapid purification method, highly concentrated and viscous dextran may disturb subsequent RNA isolation from EVs due to co‐precipitation with the reagent and interfere with EV immunolabeling by increasing artifacts from non‐specific binding of pre‐conjugated antibodies (Su et al. 2025; Yang, Zhang, et al. 2020). Adding dextranase may prevent precipitation to reduce non‐specific binding (Su et al. 2025).

Ultrafiltration using a specific molecular weight cut‐off (MWCO) membrane and external pressure can separate EVs by allowing smaller protein contaminants to pass through the membrane while retaining EVs at high throughput (Wang, Zhou, et al. 2024). It concentrates EVs swiftly and enables EV subpopulation isolation by adjusting filter size selection (Yang, Zhang, et al. 2020). Ultrafiltration yielded smaller vesicle size and better maintained integrity of *A. thaliana* EVs than PEG precipitation and ultracentrifugation (Liu et al. 2020). However, smaller‐sized EVs may be lost during filtration, and EV‐sized proteins may potentially be co‐isolated along with EVs (Fang and Liu 2022). Filter pore size selection and the pre‐centrifugation step are critical factors for ensuring selectivity, removing large debris, and avoiding EV loss (Lee et al. 2020). Using ultrafiltration as pre‐treatment purification provides superior EV yield and purity. Combining Amicon ultrafiltration (Merck) with qEV column (IZON) increased cabbage PDNV purity by 20‐fold compared to PEG precipitation and ultracentrifugation (You et al. 2021). Ultrafiltration can be used to discard the remaining unloaded substances during vesicle encapsulation (Wang, Liu, Cao, et al. 2024; You et al. 2021). Membrane pore clogging shortens its lifetime and reduces EV yield. Therefore, transitioning feed stream flow from conventional dead‐end filtration to a tangential flow system offers an innovative solution to avoid membrane fouling and enhance scalability (Yang, Zhang, et al. 2020).

TFF applies a tangential flow feed stream to the membrane surface, allowing particles smaller than the pore size to pass into the permeate stream, while retaining larger particles in the retentate stream, which returns to the feed reservoir (Paganini et al. 2019; Yang, Zhang, et al. 2020). It continuously repeats filtration to avoid membrane clogging and to upscale processing, leading to high EV yield with minimal vesicle damage (Wang, Zhou, et al. 2024; Yang, Zhang, et al. 2020). TFF had superior reproducibility and scalability than 60% iodixanol cushion ultracentrifugation during *Photinia glabra* PDNV purification and exhibited identical keratinocyte viability and senescence‐related markers after stimulation with combined TNF‐α and IFN‐γ. TFF was chosen as the preferred manufacturing method because of its superior reproducibility and scalability (Lee et al. 2025). Continuous filtration using TFF retained intact vesicles and artemisinin content of *Folium Artemisiae Argyi* PDNVs (Li et al. 2025). However, this method requires a set of equipment and a specific membrane for maintaining a consistent feed flow and continuous filtration (Giancaterino et al. 2025). Transmembrane pressure, cross‐flow velocity, and concentration factor are crucial parameters to maintain vesicle integrity and obtain the desired purity level (Zheng et al. 2025).

Dialysis is a size‐based separation via a semi‐permeable membrane bag or cassette in a buffer medium (Auquiere et al. 2025). The specific particle size of ginseng PDNVs was obtained via dialysis for further ultracentrifugation or ATPS to produce ginsenoside‐rich PDNVs (Choi et al. 2024). Selective membrane pore size can retain vesicles inside the dialysis bag and release non‐vesicular material via free diffusion (Auquiere et al. 2025). Despite isolating ginger PDNVs, hydrostatic dialysis using a 10 kDa membrane overnight failed to remove PEG6000 residue, as shown by positive barium iodide staining. Thus, optimizing membrane pore cut‐off is a necessary step for EV purification (Kalarikkal et al. 2020). Optimum dialysis time, volume, temperature, and pH of dialysis medium may prevent aggregate formation on the membrane to achieve effective purification (Auquiere et al. 2025). Applying electric current during dialysis reduces filtration time and increases selectivity via charge‐based separation. The direction of the electric current should be changed at regular time intervals to prevent clogging of the membrane pores. Electrophoretic dialysis (ELD) eliminated protein and RNA impurities from lemon PDNVs, while maintaining their homogeneous particle size and concentration (Yang, Liu, et al. 2020). However, heterogeneous surface charge and size of EVs may interfere with the efficiency of ELD (Zheng et al. 2025).

Size‐exclusion chromatography (SEC) purifies EVs from contaminants of different molecular sizes using a column packed with porous polymer microspheres. The elution rate of smaller molecules is reduced as they are able to enter the pore of the stationary phase, while larger molecules are forced to move around the outer surface of the porous material to be eluted earlier. It can preserve EV structural integrity, separate EV subpopulations, enable low‐volume sample processing, and maintain reproducibility. High‐resolution purification using SEC should consider column size, stationary phase resin and gel beads, and mobile‐phase flow rate and volume (Dilsiz 2024; Yang, Zhang, et al. 2020). Cross‐linked agarose polymer (Sepharose) is more frequently employed as a stationary phase than polyacrylamide (Sephacryl) and dextran‐agarose (Superdex) due to better EV purity and recovery (Monguio‐Tortajada et al. 2019). Sepharose SEC on pre‐ultrafiltered melon phloem solution eluted EV fractions earlier than soluble protein fractions (Sánchez‐López et al. 2024). Despite reduced EV particle concentration and yield, adding size‐exclusion chromatography after ultracentrifugation improved vesicle size homogeneity and EV marker expressions (del Pozo‐Acebo et al. 2022; López de las Hazas et al. 2023). Preserving Sepharose column using azide in PBS at 4°C may prevent contamination prior to separation (del Pozo‐Acebo et al. 2022). However, the SEC column size may limit throughput, which requires sample pre‐treatment using ultracentrifugation or ultrafiltration to concentrate the EV sample (Paganini et al. 2019). The post‐isolation concentration step should be performed after combining the eluted fractions containing EVs (Wang, Zhou, et al. 2024). Moreover, the SEC has limitations to separate EVs from impurities with a similar molecular size range (Dilsiz 2024; Yang, Zhang, et al. 2020).

Anion‐exchange chromatography (AEC) is employed to purify EVs by exploiting the interaction between the negatively charged EVs and the positively charged cation‐exchange matrix stationary phase. EVs demonstrate a negative charge due to anionic phosphatidylserine in their outer membrane (Seo et al. 2022). During ion‐based interaction, selective binding of EVs with a cation‐exchange matrix is controlled by altering ionic capacity and pH level of elution buffer to retain EVs in the column and separate them from other charged impurities. AEC offers a straightforward method with higher purity and quality than ultracentrifugation‐based and size‐based isolation (Paganini et al. 2019; Wang, Zhou, et al. 2024). AEC using an increased gradient of NaCl in HEPES buffer at pH 7.5 elevated the uniformity and purity index of cabbage and red beet PDNVs. Through measuring plant EV‐specific marker TET8 expression, most of the EV populations were eluted in medium NaCl concentration, while protein and nucleosome contaminants were found at the lower or higher NaCl fractions (Zanotti, Arena, et al. 2025; Zanotti, Troise, et al. 2025). DNA, albumin, and host cell protein also carry a negative charge and exhibit competitive binding that may reduce EV binding in the stationary phase. Thus, the pre‐treatment step is crucial for removing contaminants prior to separation (Silva et al. 2023). Ultrafiltration step prior to AEC can remove low‐mass contaminants, while diafiltration may discard high‐molecular‐weight nucleic acid or protein aggregates via buffer exchange (Zanotti, Arena, et al. 2025). Smaller bead‐size resins provide a larger surface area for EV‐binding that leads to better resolution, while the monolith convective porous matrix offers an advantage in throughput capacity by allowing a higher flow rate (Paganini et al. 2019; Silva et al. 2023). Moreover, a low‐salt‐concentration buffer may be suitable to elute pure EVs (Seo et al. 2022).

Capillary‐channeled polymer (C‐CP) implements hydrophobic interaction chromatography (HIC) via melt‐extruded polymer (nylon, polypropylene, polyester) in non‐porous micron‐sized fibers (Huang et al. 2019; Jackson et al. 2023). The space between fibre channels provide non‐fouling EV flow in a viscous mobile phase without intra‐phase hold‐up. At high salt concentration, hydrophobic EV surfaces are attached to the weakly ionized fibre surfaces. Reversing the mobile phase to a low‐salt gradient retains EVs in the stationary phase longer than proteins before elution (Huang et al. 2019). C‐CP spin‐down tips with a solid‐phase extraction mechanism yielded PDNVs from fruits and vegetables with vesicle purity above 3 × 10^10^ particles/µg protein. Moreover, recovery of plant‐origin nanovesicles was confirmed by PEN1 expression. However, the broad difference in particle concentration between plant materials was attributed to unsuitable mechanical homogenization that leads to vesicle damage and subsequently reduced NPV recovery (Jackson et al. 2023).

Affinity‐based purification utilizes specific reversible interactions, such as immunological antigen‐antibody pairings or ligand‐receptor affinities, between immobilized ligand and the EV membrane surface (Paganini et al. 2019; Wang, Zhou, et al. 2024). Targeted EV capture uses high‐affinity antibodies, aptamers, or peptides to enable specific isolation of EV subtypes, with potentially limited yield. Lipid probe, such as phosphatidylserine, is employed for non‐targeted EV capture based on the high affinity of lipids on the EV surface (Du et al. 2023; Wang, Zhou, et al. 2024). Antibody‐based approaches are the most prevalent for EV purification, either by conjugating transmembrane protein‐targeting antibodies to magnetic beads or by immobilizing them on microfluidic chips (Wang, Zhou, et al. 2024). TET8‐coated beads captured *A. thaliana* leaf EVs with non‐detected expressions of negative markers AGO2 and AGO4 (He et al. 2021). After iodixanol gradient ultracentrifugation, co‐incubation of EV‐rich fraction with TET8‐coated beads purified TET8‐positive *Nicotiana benthamiana* leaf EVs and removed co‐sedimented large vesicle impurities (Huang et al. 2021). Although this approach offers high EV purity and specificity, it may not recognize other EV subtypes with different biomarkers and lacks scalability. High‐affinity antibodies or ligands for capturing a particular type of EVs are relatively expensive, while the large size and limited mobility of magnetic beads require an extended incubation period for antibody conjugation. Low‐volume sample processing reduces EV yield for downstream analysis and clinical applications (Dilsiz 2024; Paganini et al. 2019; Yang, Zhang, et al. 2020).

Microfluidics is an integrated isolation and analysis system with parallel devices interconnected via microchannels for precise fluid control to reduce the required sample volume and vesicle damage (Dilsiz 2024; Praveena et al. 2025). It overcomes sensitivity and specificity issues found in ultracentrifugation and ultrafiltration by improving EV purity and yield, reducing processing time, and increasing throughput. Moreover, it serves as a diagnostic tool through multiplex profiling of disease‐specific markers on EVs (Kumar et al. 2023). Simultaneous injections of siRNA and grapefruit PDNVs using a non‐pulsating pump to a 3D microfluidic‐based chip loaded with siRNA into PDNVs. Injecting pressure and carrier solution are crucial factors for increasing loading efficiency and reducing particle size and dispersity (Itakura et al. 2023). However, microfluidic‐based separation requires complex integrated equipment and faces challenges with limited mass transfer and diffusion (Praveena et al. 2025).

The ultrafast isolation system applies negative‐pressure oscillation to generate dual‐frequency vibrational transverse waves toward the membrane filter. It enables label‐free purification and a clog‐free ultrafiltration system, which reduces permeate flux via an acoustofluidic stream to improve efficiency, yield, and purity. EXODUS is an automated device applying an ultrafast isolation system with excellent robustness and reproducibility (Chen et al. 2021). *Pinctada martensii* oyster mucus EV isolation using the EXODUS system was performed after pre‐centrifugation and microfiltration treatments to purify nanovesicles with detected expressions of CD9 and CD63 (Mo et al. 2025). However, the single‐channel isolation of the EXODUS system may limit its scalability, while workflow optimization is required for isolating EV subpopulations with a narrow interval. Integrating EXODUS with downstream analysis may increase processing speed and enable seamless enrichment (Chen et al. 2021).

Each isolation method has advantages and disadvantages that need to be assessed carefully. Selecting optimal NPV isolation method should consider crucial factors, including type of starting material, equipment availability, obtained NPV yield and purity, scalability and cost, and workflow and time efficiency (Table 3) (Fatima et al. 2026; Praveena et al. 2025). Ideal method should be feasible and rapid, producing high purity and yield, preserving vesicle integrity, working effectively with low‐volume sample, relatively low cost, scalable to mass production, and integrated with automatic workflow (Dilsiz 2024; Praveena et al. 2025). High NPV purity can improve the efficiency of downstream studies, while high NPV yield may ease the path toward clinical translation. The single traditional method often fails to isolate NPVs with high yield and purity in rapid and simple steps. Therefore, pairing isolation methods or incorporating emerging techniques, such as microfluidic and ultrafast isolation systems, can resolve the tradeoffs (Dilsiz 2024).

**TABLE 3:** Comparative analysis of NPV isolation methods.

Methods	NPV yield	NPV purity	NPV integrity	Scalability	Complexity	Cost	Processing time
Differential ultracentrifugation	Moderate	Low	Low	Moderate	Low	Moderate	Moderate
Density gradient ultracentrifugation	Moderate	High	High	Low	High	Moderate	Long
Polymer precipitation	High	Low	Low	High	Low	Low	Moderate
Aqueous two‐phase system	Moderate	Low	High	Moderate	Moderate	Low	Moderate
Ultrafiltration	Moderate	Moderate	Moderate	Moderate	Low	Low	Short
Tangential flow filtration	High	Moderate	Moderate	High	High	Moderate	Moderate
Dialysis	Moderate	Moderate	Moderate	Moderate	Low	Low	Long
Size‐exclusion chromatography	Moderate	High	High	Moderate	High	Moderate	Long
Anion‐exchange chromatography	Moderate	High	High	Moderate	High	Moderate	Long
Capillary‐channeled polymer	High	High	High	Low	High	Moderate	Moderate
Affinity‐based purification	Low	High	High	Low	Moderate	High	Moderate
Microfluidics	Moderate	High	High	Moderate	High	High	Short
Ultrafast isolation system	High	High	High	Moderate	Moderate	High	Short

Commercial isolation kits have become a promising recent alternative for rapidly isolating high NPV yields with minimal specialized equipment (Gandham et al. 2020). Precipitation‐based purification using ExoQuick kit (System Biosciences) after sucrose gradient ultracentrifugation elevated the purity of ginseng PDNVs and decreased the zeta potential, potentially reducing particle aggregation (Jang et al. 2022). It is not only able to isolate the majority of nanovesicles but also tends to co‐precipitate larger‐sized impurities (Jo et al. 2021). Despite a broader particle size distribution, isolation of acerola PDNVs via ExoQuick yielded a slightly higher recovery rate than the affinity‐based exoEasy kit (Qiagen). Compared with ultracentrifugation, it yielded a higher recovery rate (Umezu et al. 2021). exoEasy kit offers quick and convenient isolation by mixing the buffer with the EV‐containing solution to facilitate binding to the membrane affinity spin column (Lagos et al. 2017). Pairing the exoEasy kit with low‐speed centrifugation yielded higher yields and particle concentration of rock bream fish plasma EVs than high‐speed ultracentrifugation. However, it also exhibited cytotoxicity toward FHM and RAW 264.7 cells, possibly due to co‐isolated buffers added to the spin column prior to centrifugation (Nikapitiya et al. 2022). Overall, the EV isolation kit may yield substantial co‐isolated proteins and impurities, casting doubt on its reliability and specificity. A post‐isolation cleaning protocol should be added to remove any remaining buffers or reagents. Low‐throughput and small‐volume sample processing limit the scalability of EV isolation kits. Thus, they are not always the cost‐effective option (Dilsiz 2024; Gandham et al. 2020).

## 4 Characterization and Evaluation of NPVs

The diverse origins, morphologies, and metabolites of NPVs make it challenging to establish a standardized method for NPV characterization. A well‐developed, comprehensive characterization strategy is crucial for identifying isolated NPVs by their morphological features, metabolites, and protein markers, thereby achieving high‐quality NPV production (Hartjes et al. 2019). Choosing suitable analytical methods and instruments allows for accurate data collection and a deeper understanding of isolated NPV characteristics (Lai et al. 2022). Moreover, proper preservation methods maintain the biotherapeutic metabolites of NPVs during storage to exhibit consistent efficacy. Stability in body fluids, cellular uptake routes, biodistribution to the target, and biosafety should be assessed prior to biotherapeutic evaluation.

### 4.1 Morphological Characterization

Comprehensive physical assessment of NPVs requires, at a minimum, evaluation of particle shape, size, concentration, and zeta potential (Table 4) (Welsh et al. 2024). Particle shape analysis may distinguish EVs from non‐lipid‐bilayer impurities (Dilsiz 2024). Transmission electron microscopy (TEM) has become the standard for EV imaging via nanometer‐scale observation of EV structures to distinguish them from non‐vesicular particulates (Praveena et al. 2025; Welsh et al. 2024). TEM captures the spherical shape and lipid bilayer formation of EVs, while cryo‐TEM preserves EVs under native conditions to minimize the risk of artifact formation (Praveena et al. 2025; Webber and Clayton 2013). Negative staining using uranyl acetate prior to TEM observation visualized the enlarged lipid bilayer of lipid‐reconstructed turmeric PDNVs (Wang, Zhang, et al. 2024). Atomic force microscopy (AFM) connects the gap between imaging and physical characterization by scanning and measuring surface topography of non‐labelled EVs (Praveena et al. 2025). AFM revealed a non‐homogeneous surface of turmeric PDNVs and sesame leaf PDNVs, potentially due to protein encapsulation within the high‐density lipid membrane (Jiang et al. 2024; Liu et al. 2022). However, electron microscopy is incapable of quantifying impurity levels and may not be suitable for routine daily evaluation (Webber and Clayton 2013).

**TABLE 4:** Isolation methods and characterization results of NPVs.

Sources	Isolation methods	Mean particle size (nm)	Mean particle concentration (particles/mL)	Mean zeta potential (mV)	Identified metabolites	Ref.
*Brucea javanica*	dUC.	104.6	—	−9.2	Bruceine D, brusatol, TG.	Yan et al. (2024)
*Catharanthus roseus*	dUC; TFF; MzGU; UF.	75.51	—	−21.8	Ether‐PC, vinpocetin.	Ou et al. (2023)
*Dioscorea japonica*	dUC.	168.0	15 × 10^9^	—	Dioscorin.	Hwang et al. (2023)
Ginger	dUC; IsoGU.	294.1	—	−29.7	Digalactosyl‐DAG, gingerol, PA, shogaol.	Zhuang et al. (2015)
Ginger	dUC; IsoGU.	231.6	—	−12.0	Actin, digalactosyl‐DAG, 6‐gingerol, 6‐shogaol, PA.	Zhang et al. (2016)
Ginseng	dUC; MzGU; IsoGU.	151.6	2.24 × 10^13^	−17.9	Ceramide, ginsenosides, PC, TG.	Kim et al. (2023)
Grape	dUC.	78.8	—	−13.9	Trans‐*δ*‐viniferin, trans‐*δ*‐viniferin glycoside.	Shkryl et al. (2024)
*Lycium ruthenicum*	dUC; Prec.	114.1	4.05 × 10^9^	−6.36	Polyphenols, polysaccharides.	Zhang et al. (2024)
*Momordica charantia*	dUC.	155.0	2.5 × 10^6^	—	Ceramide, fatty acyls, TG.	Ye et al. (2024)
*Panax notoginseng*	dUC; MzGU; IsoGU.	151.3	—	−8.0	Ceramide, DUFA, MUFA, PA, PUFA.	Li et al. (2023)
*Portulaca oleracea*	dUC; IsoGU.	180.0	9 × 10^6^	−31.4	Digalactosyl‐DAG, PC, TG.	Zhu et al. (2023)
*Rehmannia glutinosa*	dUC; IsoGU.	118.0	2.6 × 10^6^	—	Flavonoids, iridoids, miR‐7972.	Qiu et al. (2023)
Rice	dUC.	139.0	10.9 × 10^10^	−17.2	Ferulic acid, γ‐oryzanol, PC, sphingomyelins, α‐tocopherol, γ‐tocopherol, γ‐tocotrienol.	Sasaki et al. (2024)
Shiitake mushroom	dUC; IsoGU.	177.6	—	−12.2	PC, PE.	Yang et al. (2025)
*Taraxacum officinale*	dUC; UF.	142.5	6.1 × 10^7^	−41.83	DGTS, PE, TAG.	Zhang et al. (2023)
Tea	dUC; IsoGU.	166.9	—	−28.8	EGCG, myricetin, PC, vitexin.	Chen et al. (2023)
Turmeric	dUC.	176.2	4.51 × 10^8^	−17.6	Demethoxycurcumin, β‐gingerone, turmeric ketone.	Wei et al. (2023)

Abbreviations: dUC, differential ultracentrifugation; DAG, diacylglycerol; DGTS, diacylglycerol trimethylhomoserine; DUFA, diunsaturated fatty acid; EGCG, epigallocatechin gallate; IsoGU, isopycnic gradient ultracentrifugation; MUFA, monounsaturated fatty acid; MzGU, moving‐zone gradient ultracentrifugation; PA, phosphatidic acid; PC, phosphatidylcholine; PE, phosphatidylethanolamine; Prec, polymer precipitation; PUFA, polyunsaturated fatty acid; TAG, triacylglyceride; TFF, tangential flow filtration; TG, triglyceride; UF, ultrafiltration.

Nanoparticle tracking analysis (NTA) detects individual EVs via Brownian motion and measures their size and concentration. It offers high‐resolution size distribution data and facilitates fluorescence labelling detection (Dilsiz 2024; Praveena et al. 2025). Dynamic light scattering (DLS) provides quick, direct EV particle size measurement by focusing a laser on the EV solution to analyse the time‐dependent intensity of the scattered light. However, the formation of large particles may interfere with the scattering signal, leading to an inaccurate size distribution (Praveena et al. 2025). Particle size measurements using NTA and DLS in the hydrodynamic state appear larger than those using TEM due to particle swelling (Chen, Li, et al. 2022, Zhu et al. 2023). A narrow particle‐size range and a high‐sensitivity instrument can improve the precision and accuracy of particle concentration measurements (Gupta et al. 2021).

Electrophoretic light scattering (ELS) measures zeta potential to analyse the surface charge of EVs. Their membrane contains anionic lipids and proteins that drive their zeta potential to a negative value. The integration of DLS and ELS into a single instrument enables the rapid and straightforward assessment of the particle size and zeta potential of EV suspensions (Midekessa et al. 2020; Park et al. 2026; Seo et al. 2022). A zeta potential value around −20 mV is suggested as a stable value for EVs to form stable suspensions with less particle aggregation and to favour cellular absorption (Kim, Jung, et al. 2020). Ginger PDNVs fraction with low zeta potential value demonstrated high stability in gastrointestinal fluids (Zhang, Viennois, et al. 2016). Nano flow cytometry (NanoFCM) provides size, concentration, and surface biomarker analyses of EVs using bead‐based labelling detection (Dilsiz 2024). NanoFCM enabled particle concentration measurement with label‐free detection of garlic PDNVs (Wang, Liu, Dong, et al. 2024). Luminal enzymatic activity assay using fluorescein diacetate could evaluate membrane integrity of marine algae *Tetraselmis chuii* EVs using a spectrofluorometer. Fluorescein diacetate can penetrate phospholipid bilayers and is subsequently hydrolyzed by esterases inside the vesicles to produce a negatively charged green fluorescent molecule (Adamo et al. 2025).

### 4.2 Protein Marker Identification

Characterizing integrated protein markers on EVs is an important step toward understanding their biogenesis and protein function (Gandham et al. 2020). Protein marker analysis of NPVs remains challenging due to the lack of standardized guidelines for plant‐origin EVs, lower protein content than mammalian‐derived EVs, and interspecies variability (Li et al. 2024). MISEV 2023 recommends identifying at least one protein from three categories of transmembrane or GPI‐anchored proteins, cytosolic proteins recovered in EVs, and major components of non‐EV co‐isolated structures (Welsh et al. 2024). To minimize the influence of other EV protein contents, markers should be detected in their native state rather than from overexpression (Pinedo et al. 2021). Immunoblotting is a common method to identify and quantify specific marker proteins of EVs through denaturation, protein separation, and antibody detection. Despite its selectivity and simplicity, it has relatively low throughput and cannot detect ligand‐antigen interactions. Enzyme‐linked immunosorbent assay (ELISA) provides a high‐throughput, sensitive method for quantitative protein analysis and ligand‐antigen detection by capturing protein on a solid‐phase carrier prior to detection with a secondary antibody. However, it requires a long processing time and specialized equipment (Praveena et al. 2025; Wang, Zhou, et al. 2024).

Tetraspanins are transmembrane‐bound proteins in EVs associated with membrane trafficking and biosynthetic maturation (Shao et al. 2018). Although mammalian‐origin tetraspanins, such as CD9, CD63, and CD81, are often used to characterize NPV purity, this may lead to inaccurate detection compared to natural source‐specific markers. Ideal NPV protein markers should be naturally enriched and conserved across species (Pinedo et al. 2021). Plant‐derived tetraspanin 8 (TET8) is a highly suitable candidate, since it has a similar structure to CD63 and is highly enriched in *A. thaliana* EVs (Koch et al. 2025; Pinedo et al. 2021). Moreover, it is natively detected in several NPVs, such as grape, tobacco, and broccoli EVs (He et al. 2021; López de las Hazas et al. 2023; Shkryl et al. 2024). Plant syntaxins PEN1 and PEN3 are also proposed as candidates (Jackson et al. 2023; Koch et al. 2025; Zhang, Qiu, et al. 2020). Cytosolic proteins, such as HSP70, have also been identified in both plants and mammalian EVs (Bai et al. 2024). Endosomal sorting complex required for transport (ESCRT)‐complex members (TSG101, Alix), pattelins, and annexins were detected in various plant species (Table 5) (Bai et al. 2024; Gandham et al. 2020; Pinedo et al. 2021). Endoplasmic reticulum and nucleus proteins, such as calnexin, GP96, and histones, are used as negative markers to determine minimum contamination from cellular debris (Jokhio et al. 2024; Newman et al. 2022; Webber and Clayton 2013).

**TABLE 5:** Current protein markers and their functions for NPV identification.

Category	Functions	Protein markers	Identified NPV sources	Ref.
Endosomal sorting complex required for transport (ESCRT)	Facilitating biosynthesis and release of NPVs.	TSG101, Alix.	Maca, ginger, bitter melon, *A. thaliana*.	Hong et al. (2023); Jokhio et al. (2024); Pinedo et al. (2021)
Heat shock protein	Assisting protein folding, stabilization, and transportation.	HSP70.	Garlic, Grape, *A. thaliana*.	Bai et al. (2024); Pinedo et al. (2021); Shkryl et al. (2024)
Patellin	Involving in plant development and stress tolerance regulation.	PATL1.	*A. thaliana*.	Koch et al. (2025); Pinedo et al. (2021)
Pentacyclic triterpene synthase	Signalling stress and defense of the plant immune system.	PEN1, PEN3.	*A. thaliana*, *N. benthamiana*.	Jackson et al. (2023); Koch et al. (2025); Zhang et al. (2020)
Protein argonaute	Involving in RNA‐mediated gene silencing.	AGO1, AGO2.	*A. thaliana*.	Pinedo et al. (2021); Koch et al. (2025)
RPM1‐interacting protein	Regulator for plant immunity.	RIN4.	*A. thaliana*.	Koch et al. (2025)
Tetraspanin	Essential for inter‐species communication and cell trafficking.	CD9, CD54, CD63, CD81, TET8, TET9.	Garlic, grape, grapefruit, bitter melon, *A. thaliana*.	Jokhio et al. (2024); Koch et al. (2025); Pinedo et al. (2021); Savcı et al. (2021)

Abbreviations: AGO, protein argonaute; CD, cluster of differentiation; HSP70, heat shock proteins 70 kDa; PATL1, patellin 1; PEN, pentacyclic triterpene synthase; RIN4, RPM1‐interacting proteins; TET, tetraspanins; TSG101, tumor susceptibility gene 101.

Protein markers can also identify the organelle origin of EVs. Expression of ole e 1 and 12 in olive pollen EVs indicated their origin from the endoplasmic reticulum of germinated pollen (Prado et al. 2014). H^+^/ATPase is a specific marker for plant and protist membrane proteins, showing positive expression in the *T. chuii* EVs (Adamo et al. 2021; Paterna et al. 2022). It is worth noting that no specific marker that is expressed only for EVs. Common EV markers, such as tetraspanins, can be expressed on other subcellular organelles simultaneously Yang, Zhang, et al. 2020). Although specific NPV protein markers are not yet clearly established in the guidelines, careful selection of native markers based on their organelle origin and species can determine their identity and biogenesis (Dad et al. 2021; Li et al. 2024).

### 4.3 Metabolites Analysis

NPVs carry endogenous metabolites from their sources, including lipids, proteins, nucleic acids, and secondary metabolites (Tables 4 and 6). Proteomic analysis on EVs by LC‐MS/MS can be performed using untargeted and targeted workflows for qualitative screening of available proteins and quantitative assessment of specific protein abundance, respectively (Newman et al. 2022). Mass spectrometry offers high‐throughput, sensitive analyses with or without labelling, although it requires laborious sample preparation and considerable processing time (Shao et al. 2018). Total protein quantification using the bicinchoninic acid (BCA) assay is commonly used for its versatility and compatibility with various samples, whereas the Bradford assay offers greater time efficiency and sensitivity (Zhang, Bi, et al. 2020). However, both assays are interfered with protein contaminants from improper EV isolation, which may cause overestimation of EV populations (Du et al. 2023; Gupta et al. 2021). Every 1 µg of EV protein may approximately correspond to 2 × 10^9^ EV particles (Sverdlov 2012). EV purity can be determined by calculating the ratio between particle and protein concentrations (Lai et al. 2022). The ratio above 3 × 10^10^ EV particles/µg protein may represent high EV purity, the ratio of 2 × 10^9^ ‒ 2 × 10^10^ delineates low EV purity, and EVs with a ratio below 1.5 × 10^9^ is considered impure (Webber and Clayton 2013).

**TABLE 6:** Therapeutic mechanism of NPVs toward various disease model experiments.

Sources	Identified metabolites	Disease models	Cell and animal models	Mechanism of actions	Ref.
*Brucea javanica*	Bruceine D, brusatol, TG.	Breast cancer.	4T1 cells. TNBC mice.	Activated apoptosis via PI3K/AKT/mTOR pathway.	Yan et al. (2024)
*Dioscorea japonica*	Dioscorin.	Osteoporosis.	MC3T3‐E1 cells. OVX‐induced mice.	Activated osteoblast differentiation and proliferation via BMP‐2/p‐p38‐dependent Runx2 pathway.	Hwang et al. (2023)
Ginger	Digalactosyl‐DAG, monogalactosyl‐MAG, PA, 6‐shogaol.	Hepatic steatosis.	Alcohol‐treated mice.	Activated Nrf2 in hepatocytes via the TLR4/TRIF pathway.	Zhuang et al. (2015)
Ginseng	Ceramide, PC, TG.	Glioma.	3T3‐C6 cells. C6 Glioma orthotopic rats.	Penetrated the BBB and suppressed M2 polarization.	Kim et al. (2023)
Ginseng	Ginsenosides Rb1 and Rg1.	Osteoporosis.	BMM cells. LPS‐treated mice.	Inhibited osteoclast differentiation via actin reduction and AKT activation.	Seo et al. (2023)
Ginseng	Ginsenosides Rg1, Re, Rh1, Rg2, Ro, and Rd.	Topical wound.	HaCaT, HUVEC, and BJ cells.	Enhanced keratinocyte migration and HUVEC angiogenesis via ERK and AKT/mTOR pathways.	Yang et al. (2023)
*Lycium barbarum*	Cellobiose, D‐fructose, glucose, maltose, D‐mannose.	Muscle atrophy.	C2C12 cells. Dexamethasone‐treated mice.	Activated AMPK/SIRT1/PGC1α pathway and alleviated myosin heavy chain isoform transition.	Zhou et al. (2024)
*Momordica charantia*	Ceramide, fatty acyls, TG.	Cardiotoxicity.	H9c2 cells. Doxorubicin‐treated mice.	Suppressed the degradation of p62 ubiquitination via promoting Nrf2 nuclear translocation.	Ye et al. (2024)
*Morus nigra*	Kaempferol‐3‐*O*‐glucoside, quercetin‐3‐*O*‐glucoside, rutin.	Hepatocellular carcinoma.	Hepa1‐6 cells. Orthotopic liver cancer mice.	Induced apoptosis via G0/G1 phase arrest and triggered intracellular ROS.	Gao et al. (2024)
*Pueraria lobata*	Puerarin.	Post‐menopausal osteoporosis.	Human bone marrow stem cells. TMAO‐treated rats.	Promoted osteogenesis via TMAO degradation for osteoblast differentiation and bone mineralization.	Zhan et al. (2023)
*Rehmannia glutinosa*	miR‐7972.	Lung inflammation.	LPS‐treated mice.	Induced M2 macrophage polarization and downregulated GPR161.	Qiu et al. (2023)
Rice	PC, PE, PS, sphingomyelins.	Colon cancer.	Colon26 cells. Colon26‐fluc‐transplanted mice.	Induced apoptosis via DNA fragmentation and chromatin condensation.	Sasaki et al. (2024)
*Taraxacum officinale*	DAG, PE, TG.	Chronic hypertension.	Intermittent hypoxic mice.	Inhibited systemic inflammation and vascular wall remodelling by increasing butyrate production.	Zhang et al. (2023)
Tea	EC, ECG, EGCG, myricetin, vitexin.	Breast and lung cancer.	MCF‐7 and 4T1 cells. Breast xenograft tumor mice.	Induced apoptosis and cell cycle arrest via mitochondrial damage.	Chen et al. (2022)
Turmeric	Curcumin, DAG, PC, PE, TAG.	Ulcerative colitis.	DSS‐induced colitis mice.	Promoted M2 polarization to inhibit CD16/32 and elevate CD206.	Gao et al. (2022)

Abbreviations: BBB, blood brain barrier; BMM, bone marrow‐derived macrophages; DAG, diacylglycerol; DSS, dextran sulfate sodium; EC, epicatechin; ECG, epicatechin gallate; EGCG, epigallocatechin gallate; Fluc, firefly luciferase; HUVEC, human umbilical vein endothelial cells; LPS, lipopolysaccharide; PA, phosphatidic acid; PC, phosphatidylcholine; PE, phosphatidylethanolamine; PS, phosphatidylserine; MAG, monoacylglycerol; OVX, ovariectomized; ROS, reactive oxygen species; TAG, triacylglyceride; TG, triglyceride; TMAO, trimethylamine *N*‐oxide; TNBC, triple negative breast cancer.

NPVs also inherit nucleic acid materials from their sources to engage intercellular communication and gene expression in target cells (Fang and Liu 2022; Zou et al. 2024). Bioinformatics analysis using gene and genomic annotation pathway (KEGG) and Gene Ontology (GO) databases can predict their potential bioactivity mechanism (Jiang et al. 2024; Li et al. 2023). *Crassostrea hongkongensis* oyster nanovesicles with obtained purity of 8.75 × 10^9^ particles/mg protein and yield of 3.14 × 10^9^ particles/g oyster had 3468 identified proteins via LC‐MS/MS label‐free quantitative proteomics. GO analysis showed that the most enriched proteins were involved in binding, catalytic activity, organismal systems, cellular processes, and metabolic processes. Further KEGG pathway analysis of the top 20 proteins suggested osteogenic differentiation‐related signalling pathways, supporting their potential to regulate bone homeostasis (Hu, Hou, Liu, et al. 2024).

EV membranes are composed of three major lipid classes of phosphoglycerolipids, sterols, and sphingolipids, along with the incorporated peripheral membrane proteins. They provide favourable lipid bilayer curvature and facilitate receptor interaction at target sites, thereby transporting bioactive molecules and inducing specific cellular functions (György et al. 2015; Tenchov et al. 2022). Their lipid compositions may vary across species, while variations during juice extraction from sources may contribute to inconsistent lipid class composition within a single species (Zhou et al. 2023). Lipid quantification using LC‐MS/MS is preferred over the colorimetric method, such as the sulfophosphovanilin assay, due to high sensitivity and specificity (Gupta et al. 2021). Lipidomic analysis of sesame leaf PDNVs using LC‐MS/MS revealed ether‐linked diacylglycerol as the primary lipid constituent, along with triglyceride, diacylglycerol, phosphatidylethanolamine, and ceramide (Jiang et al. 2024). Comparative lipidomics on each nanovesicle from marine species of *Patinopecten yessoensis* scallop, *Meretrix* *meretrix* clam, and *C. hongkongensis* oyster exhibited the highest lipid content of phosphatidylserine (49.25% of lipids), phosphatidylethanolamine (26.4%), and phosphatidylcholine (24.8%), respectively (Hu, Hou, Liu, et al. 2024; Hu, Hou, Quan, et al. 2024; Hu, Quan, et al. 2024).

Secondary metabolites from origin sources are naturally encapsulated in NPVs during biogenesis. They are carried within NPVs and subsequently released into target sites to trigger biomedical effects. The ability of turmeric PDNVs to naturally carry curcumin was demonstrated by the detection of curcumin synthase proteins and enzymes using LC‐MS and RT‐qPCR, respectively (Wei et al. 2023). Similarly, ginseng PDNVs carry ginsenosides Rg1, Re, Rh1, Rg2, Ro, and Rd to contribute for wound healing (Yang, Lu, et al. 2023). Selective isolation methods can enrich specific phytochemicals in NPVs. For example, sucrose gradient ultracentrifugation of ginger PDNVs yielded two fractions with distinct metabolite profiles, despite similar physical properties. The shogaol‐rich fraction was obtained from the interface between the 8% and 30% sucrose layers, while the gingerol‐rich fraction floated between the 30% and 45% sucrose layers (Zhang, Viennois, et al. 2016; Zhuang et al. 2015).

### 4.4 Storage and Preservation

EV stability during preservation is crucial to maintain the biofunctional properties of their components before delivery to the target site, thereby triggering subsequent biomedical effects. Maintaining optimum storage temperatures, pH levels, media, and techniques is pivotal in preservation. Storing EVs at −80°C is the recommended method to preserve their structural characteristics and vesicle components for maintaining consistent bioactivity (Praveena et al. 2025; Welsh et al. 2024). However, extended storage periods at −80°C after prolonged time may reduce EV concentration, increase minor protein contents due to EV lysis, enlarge particle size, and shift the zeta potential toward a positive value due to EV aggregation and membrane phospholipid rearrangement (Gelibter et al. 2022; Maroto et al. 2017). Thus, adding cryoprotective agents, such as trehalose and dimethyl sulfoxide (DMSO), can reduce potential damage to vesicle (Bosch et al. 2016; Gelibter et al. 2022). Moreover, this storage condition is energy‐intensive, difficult to transport, and relatively infeasible for clinical application (Trenkenschuh et al. 2022). Frequent freeze‐thaw cycles may increase particle size and reduce EV concentration, which can lead to membrane disruption and particle aggregation. Fresh EVs should be preferred for bioactivity studies to ensure consistency and reliability of results (Gelibter et al. 2022; Praveena et al. 2025).

Freeze‐drying or lyophilization offers a feasible, prolonged EV stabilization approach to facilitate robust long‐term analyses and precise clinical translations. Lyophilized EVs can achieve colloidal stability for several months at 2‒8°C (Trenkenschuh et al. 2022). Its workflow consists of freezing the EV solution to −50°C, increasing the temperature for primary drying to −20°C, elevating the temperature for secondary drying to 20°C, and holding this condition for 8‒12 h before releasing the vacuum (Susa et al. 2023; Trenkenschuh et al. 2022). Removal of water content from the EV sample yields a solid EV formulation, which is considered more stable than the liquid form (Guarro et al. 2022; Susa et al. 2023). During lyophilization, EVs may experience stress due to ice crystal formation, buffer salt precipitation, increased ionic strength, and particle swelling, which can induce vesicle damage (Trenkenschuh et al. 2022). Adding lyoprotectant, such as sucrose or trehalose, is crucial to maintain vesicle structure and prevent aggregation. They can interact with phospholipid head groups in EV membrane to prevent ice crystal formation, while their high viscosity and high transition temperature nature prohibit vesicle leakage and crystallization (Guarro et al. 2022).

Stability evaluation is performed by measuring characterization parameters after preservation under various conditions and durations, as minimal change in parameter values is desired. Storing grape PDNVs at −80°C in PBS preserved their cup‐shaped and lipid bilayer structure after six months. Moreover, adding trehalose during the accelerated freeze‐thaw experiment minimized the rate of their particle number reduction (Zheng et al. 2026). After seven days of storage at below 60°C, luteolin‐loaded sesame leaf PDNVs retained initial particle size and zeta potential values and showed more than 80% luteolin (Jiang et al. 2024). Lyophilization using trehalose on Exocomplex, a fruits and vegetables PDNV preparation, did not significantly change their particle size and concentration profiles (Di Raimo et al. 2023).

Light exposure may induce photo‐oxidation of unsaturated fatty acids and degradation of EV phospholipid (Yang et al. 2025). Sesame leaf PDNVs exhibited a greater ability to minimize the rate of luteolin degradation than free luteolin under continuous exposure to 365 nm UV light, despite a gradual reduction in luteolin after 72 h of exposure (Jiang et al. 2024). Shiitake mushroom PDNVs showed higher particle dispersity than the liposome preparation under 300 lux light exposure after 30 days (Yang et al. 2025). Therefore, it is essential to prevent light exposure during EV storage to reduce their changes.

Incubation of sesame leaf PDNVs in NaCl solution altered their zeta potential, suggesting particle aggregation, possibly due to reduced electrostatic repulsion relative to hydrophobic interactions and van der Waals forces. Preserving sesame leaf PDNVs at neutral pH prevented particle size and zeta potential from increasing compared to incubation in an acidic environment (Jiang et al. 2024). *C. roseus* PDNV particles were divided, merged, and enlarged with surfactants, strong acid, and strong base incubation, respectively (Ou et al. 2023). Adding Saliguard TMO, a paraben‐free preservative, to *Dendropanax morbifera* leaf PDNVs reduced the proportion of enlarged particles during four weeks of storage (Kim et al. 2022). Establishing suitable storage and preservation protocols for NPVs is essential to maintain morphological stability, preserve intrinsic components, and sustain beneficial effects for effective and reliable clinical applications (Bai et al. 2024).

### 4.5 Stability Screening in Digestive Systems

The harsh nature of body fluids is a significant obstacle to the NPV distribution and targeting. NPV stability must be assessed using enzymatic simulation and incubation in gastrointestinal fluids to mimic the actual environment during transportation to the target site. A minimum shift in characteristic parameters after treatment is preferred to indicate a sustained EV structure. The integrated lipid‐bilayer membrane of NPVs protects encapsulated therapeutic materials, resists external degradation, and preserves vesicle integrity (Pomatto et al. 2023).

Several studies have demonstrated the stability of NPVs in various body fluids and enzymatic digestion environments. Shiitake mushroom PDNVs exhibited better stability than liposomes as shown by negligible changes in particle size and lower dispersity after incubation at pH 2‒12 (Yang et al. 2025). In gastrointestinal model using simulated gastric (pH 2.0) and intestinal (pH 6.5) fluids, the surface charge of ginger and biyang floral mushroom PDNVs shifted from negative (−) to positive (+) in gastric fluid, then returned to negative (−) in intestinal fluid. Thus, both PDNVs adapted to body fluids and showed favourable absorption in the intestine (Ling et al. 2024; Zhuang et al. 2015). *C. roseus* PDNVs appeared unaffected by enzymatic digestion, since their particle size was not altered by RNase, DNase, or proteinase K (Ou et al. 2023).

### 4.6 Cellular Uptake

EVs enter cells via endocytosis through the sequential steps of vesicle formation with cargo, endosomal delivery inside the cell, and intracellular vesicle distribution (Li et al. 2024). The capability of EVs to engage in diverse endocytic pathways can dissipate intracellular energy and enhance cellular uptake (Xiao et al. 2022). In addition, phospholipids in NPVs facilitate efficient target delivery into cells (Gao et al. 2024). Endocytosis occurs through several specific pathways, including clathrin‐dependent endocytosis, caveolin‐dependent endocytosis, phagocytosis, and macropinocytosis. The cellular uptake route can be determined by co‐incubating NPVs with target cells in the presence of endocytosis inhibitors. Each inhibitor targets a specific endocytosis pathway and disrupts NPV uptake, indicating the specific pathway involved (Ling et al. 2024; Zhuang et al. 2015).

*Curcumae Rhizoma* PDNV uptake by Caco‐2 cells was inhibited by β‐cyclodextrin (a caveolin‐dependent inhibitor) and chlorpromazine (a clathrin‐dependent inhibitor), but not by amiloride (a macropinocytosis inhibitor) or quercetin (a clathrin‐ or caveolin‐independent inhibitor), indicating a selective caveolin‐ and clathrin‐dependent uptake mechanism (Yang, Peng, et al. 2023). Two fractions of ginger PDNVs obtained by sucrose gradient ultracentrifugation exhibited different endocytosis pathways, possibly due to differences in their phytochemicals. Shogaol‐rich PDNV uptake was disrupted by amiloride, indicating macropinocytosis, whereas nocodazole hindered gingerol‐rich PDNV uptake, suggesting a microtubule polymerization‐dependent pathway (Zhuang et al. 2015). Low temperature and colchicine may reduce cellular internalization of NPVs (Kim et al. 2023).

Lipophilic PKH dyes, such as PKH26 and PKH67, are commonly used to trace cellular uptake of EVs due to their simple labelling protocol and satisfactory signal stability (Melling et al. 2022; Rashidi et al. 2025; Yang, Peng, et al. 2023). However, PKH dyes tend to aggregate and form micelle artifacts due to co‐precipitation during ultracentrifugation. Their aggregate can be taken up by cells, leading to false‐positive observations of cellular internalization (Bao et al. 2023; Lai et al. 2015). Using other dyes, such as DiR, and novel lipophilic labelling techniques provide alternatives for detecting cellular uptake. After membrane labelling, avoiding ultracentrifugation and performing a thorough wash step are critical for obtaining accurate observations (Bao et al. 2023; Zhuang et al. 2015).

### 4.7 In Vivo Biodistribution

The transportation and distribution of NPVs across physiological barriers to accumulate at target sites are crucial for determining therapeutic efficacy. Biodistribution of NPVs is influenced by species differences, routes of administration, nanovesicle structure, and ability to cross physiological barriers (Fang and Liu 2022). Carbonaceous dyes, like DiD, DiI, DiO, and DiR, are favored for EV staining in vivo because they emit long near‐infrared wavelengths, which help reduce autofluorescence in animal tissues, thereby improving image clarity and contrast. PKH dyes are less commonly used due to their shorter wavelengths (Bao et al. 2023; Boudna et al. 2024). Developing an efficient in vivo fluorescence labelling strategy can improve the observation of NPV biodistribution.

Identifying administration routes with high efficacy and efficient biodistribution at the target site supports seamless bioactivity studies and clinical application. Blood‐brain barrier (BBB)‐mimicked Transwell experiments can be used to predict BBB penetration. Lipophilic membrane staining with DiI was used to label maca PDNVs as they crossed the BBB and distributed to the brain (Hong et al. 2023). Intracranial administration of DiD‐labelled ginseng PDNVs exhibited more effective glioma size reduction than intravenous administration, since it avoided degradation by metabolic components (Kim et al. 2023).

Oral administration of DiR‐labelled black mulberry PDNVs reached peak level in the gastrointestinal tract after 24 h and was maintained over 72 h. They mainly accumulated in the jejunum and colon mucosa of mice, suggesting these as the major sites of absorption before further accumulation in liver tumours via galactose receptor‐mediated targeting (Gao et al. 2024). Various administration routes of *C. roseus* PDNVs were tested to determine effective distribution. Despite their high stability in gastric juice upon oral administration, intraperitoneal injection resulted in the best absorption and distribution to pulmonary and abdominal organs, without accumulation in the brain. Distribution via tail vein injection was limited to the upper abdomen and extremities, whereas intravenous injection was rapidly cleared by the liver and spleen (Ou et al. 2023).

### 4.8 Biosafety Evaluation

Biosafety is a crucial factor in clinical translation of nanomedicines, since an ideal drug delivery system must be non‐toxic, non‐immunogenic, and have minimal side effects (Dad et al. 2021; Gao et al. 2024). NPVs are considered highly safe and biocompatible delivery platforms because natural products have been consumed with relatively low toxicity (Fang and Liu 2022). Biosafety can be assessed in vivo by evaluating organ toxicity using related parameter measurements. For instance, body weight and organ index in mice were unaffected by oral gavage of tea leaf PDNVs. While intravenous injection showed potential hepatotoxicity, as evidenced by reduced body weight, increased liver and spleen indices, and abnormal liver serum levels (Chen, Li, et al. 2022; Chen et al. 2023). After accumulation in the liver, oral intake of *Dioscorea japonica* PDNVs did not alter liver‐related parameters (serum ALT, liver triglyceride, glucose, and total protein levels) or kidney‐related parameters (BUN and serum creatinine levels) in both male and female mice after 7 days (Hwang et al. 2023). However, these studies are regarded as short‐term in vivo models and lack comprehensive clinical safety evidence. Additionally, incomplete identification and quantification of bioactive components lead to inaccuracies in understanding their mechanism of action (Zheng et al. 2025). Further clinical biosafety studies in humans are needed to standardize immunogenicity, long‐term toxicity, and pharmacokinetics for appropriate clinical dosing regimens (Wang, Zhou, et al. 2024; Zheng et al. 2025).

## 5 In Vitro and in Vivo Bioactivity Assessment of NPVs

Previous research on NPV potential and pathways for treating various diseases should underpin the target bioactivity and the design of assays. Biotherapeutic efficacy, efficiency, and safety must be assessed through both in vitro and in vivo experiments (Table 6). Biotherapeutic evaluation of NPVs requires a comprehensive strategy to accurately determine biomedical effects, appropriate dosage for clinical trials, and rigorous validation of the beneficial claim.

### 5.1 Anti‐Cancer Treatment

The nano‐sized lipid bilayer structure of NPVs can enhance penetration into target tumours, improving efficacy compared to synthetic nanovesicles and drugs alone. Highly aggressive triple‐negative breast cancer growth and metastasis were inhibited by *Brucea javanica* PDNVs by increasing pro‐apoptotic cleaved caspases 3 and 9, and reducing the expression of p‐PI3K, p‐AKT, and p‐mTOR, thereby activating apoptosis in 4T1 cells through the PI3K/AKT/mTOR pathway. The level of the breast cancer marker CA153 decreased, suggesting slowed cancer progression. Additionally, the reduction in VEGF and CD31 levels indicates anti‐angiogenic activity that helps limit tumor growth and metastasis (Yan et al. 2024).

Tea has long been recognized as an antioxidant, and tea‐derived PDNVs contain beneficial phytochemicals for cancer therapy. Tea flower PDNVs exhibited selective toxicity, targeting tumor cells while sparing normal human HUVEC and HEK293T cells. MCF‐7 and NT1 cells exhibited cell‐cycle arrest due to suppressed mitosis and DNA replication, linked to decreased levels of cyclins A and B. Additionally, elevated production of ROS, nitric oxide, and superoxide anions led to mitochondrial damage in both cell types. Intravenous delivery was more effective than oral delivery in reducing breast tumor growth and lung lesions, primarily through increased apoptosis, without adversely affecting mouse survival (Chen, Li, et al. 2022). Rice bran EVs displayed higher inhibition on colon cancer proliferation than doxorubicin HCl liposome (Doxil). The cell cycle of Colon26 cells was disrupted by reducing the G1 and S phases, increasing the G2/M phase, and inducing apoptosis via DNA fragmentation. Treated mice showed minimal luminescence of transplanted luciferase‐expressing Colon26 cells (Sasaki et al. 2024).

Glioma, a fatal brain cancer with only a 6.8% survival rate, requires challenging selective treatment to cross the BBB. Pairing differential ultracentrifugation with sucrose cushion and gradient ultracentrifugation was employed to purify ginseng PDNVs, which penetrated the BBB after intravenous injection for facilitating intra‐tumoral accumulation via macropinocytosis and GLUT‐1 endocytosis pathways in C6 glioma cells. Glioma cell growth was inhibited through suppressing M2 polarization, altering macrophage morphology, and decreasing CD206 and IL‐10 expressions, shown by reduced luminescence of orthotopic C6‐luciferase (Kim et al. 2023).

Metabolite content in NPVs may play a pivotal role in their observed bioactivities. The galactose groups on black mulberry leaf PDNVs facilitated internalization into hepatoma cells, leading to G0/G1 phase arrest, ROS induction, and apoptosis. Oral administration exhibited selective targeting toward liver tumours and modulation of gut microbiota without damaging healthy liver tissue (Gao et al. 2024). Ceramides and proteins of ginseng PDNVs facilitated TLR4‐ and MyD88‐dependent M1 macrophage polarization, inhibiting melanoma growth. Melanoma‐associated macrophages were targeted by increasing M1‐related cytokines (IL‐6, IL‐12, TNF‐α) and limiting M2‐related CD8^+^ T proliferation (Cao et al. 2019).

### 5.2 Anti‐Inflammatory Effect

*Rehmannia glutinosa* PDNVs carried miR‐7972, which can switch M1 to M2 macrophage polarization via pro‐inflammatory cytokines, NO, and ROS reduction during LPS stimulation. miR‐7972 bound to the core sites of GPR161 to activate the Hedgehog signalling pathway in lung tissue through SHH and Ptch1 upregulations, as well as SMO and Gli1 downregulations. Notably, *Escherichia coli* biofilm formation was inhibited by targeting stx2 expression, thereby reshaping the composition of the gut microbiota (Qiu et al. 2023). Green tea leaf‐derived *L. plantarum* EVs increased CD14 and ICAM‐1 expressions to promote THP‐1 differentiation. Hyper‐inflammatory skin conditions were reduced by boosting M2 polarization in THP‐1 macrophages, increasing M2b‐specific factors without significantly affecting M2a and M2c factors (Kim, Lee, et al. 2020).

Ulcerative colitis, a non‐specific inflammatory bowel disease, usually manifests as abdominal pain, diarrhea, and hematochezia that may increase the risk of colorectal cancer. Intragastric administration of garlic PDNVs alleviated DSS‐induced colitis symptoms, including body weight loss, colon shortening, hematochezia, and diarrhea, by inhibiting pro‐inflammatory cytokines and mitigating gut dysbiosis. Garlic PDNVs diminished colon lesion damage by reducing inflammatory cell infiltration, crypt destruction, and goblet cell loss through the suppression of TNF‐α and IL‐6 expression. Moreover, the *Firmicutes*/*Bacteroidetes* ratio in mice with colitis was restored to a normal state after treatment (Wang, Liu, Dong, et al. 2024). Turmeric PDNVs carried curcuminoids, including curcumin, demethoxycurcumin, and bisdemethoxycurcumin, and alleviated colitis by inducing M2 macrophage polarization, inhibiting CD16/32, and increasing CD206 in the colon lamina propria (Gao et al. 2022).

*Portulaca oleracea* PDNVs increased the abundance of the probiotic *Lactobacillus reuteri* in the human gut, thereby inhibiting the colonization of pathogenic bacteria, exhibiting immunomodulatory effects, and enhancing gut barrier integrity. Those alterations contributed to colitis mitigation by promoting the differentiation of CD4^+^CD8^+^ T cells following AhR activation and Zbtb7b downregulation (Zhu et al. 2023). Phosphatidylcholine isoform 1,2‐DLPC was identified as an active anti‐inflammasome component in garlic chive PDNVs. Oral administration of garlic chive PDNVs effectively inhibited NLRP3 inflammasome assembly and activation, suppressing chronic inflammation without affecting the health of obesity‐induced mice (Liu et al. 2021).

### 5.3 Attenuating Metabolic Diseases

A high‐fat diet leads to potential health problems, such as obesity and type 2 diabetes. AhR overexpression has been suggested to inhibit the insulin response during glucose intake. Ginger PDNVs restored gut epithelial homeostasis by targeting AhR inhibition through miR‐375 induction, which binds to the AhR 3′ UTR. Moreover, VAMP7 reduced the intracellular level of miR‐375 to maintain AhR suppression. This action reduced AhR‐induced metabolic disruption, thus preventing obesity and insulin resistance (Kumar et al. 2021). Phosphatidic acid (36:4) in garlic PDNVs significantly reduced insulin resistance by inhibiting c‐Myc activity in brain microglial cells during chronic low‐grade inflammation. Activation of c‐Myc increases mitochondrial membrane permeability, leading to mitochondrial DNA leakage and subsequent activation of the cGAS/STING inflammatory pathway. Garlic PDNVs entered microglial mitochondria to prevent mitochondrial DNA leakage and cGAS/STING activation. Microglial cell activity was modulated by suppressing the IDO1‐AhR signalling pathway, thereby enhancing glucose tolerance and insulin response sensitivity (Sundaram et al. 2022).

Mung bean sprout PDNVs lowered blood glucose levels during the treatment of type 2 diabetes via the PI3K/AKT pathway activation to regulate AKT Ser/Thr kinase and accelerate GLUT4 transport from the cytoplasm to the cell membrane. Protective effects on the liver and pancreas of HFD/STZ‐induced mice were observed, including reduced hepatocyte inflammatory infiltration, decreased lipid vacuolization, and increased pancreatic islet cell population (He et al. 2023). Ginger PDNVs demonstrated the ability to deliver 6‐shogaol to target mouse hepatocytes during alcohol‐induced liver injury treatment. Accumulation in the livers of alcohol‐fed mice was achieved within 10 min of oral administration, effectively reducing fat accumulation and inhibiting hepatic steatosis. Stimulation of hepatocytes with 6‐shogaol‐loaded ginger PDNVs increased nuclear Nrf2 and reduced ROS production. This Nrf2 activation was not observed in TLR4‐ or TRIF‐knockout mice hepatocytes, suggesting that the TLR4/TRIF pathway mediated Nrf2 activation (Zhuang et al. 2015).

### 5.4 Relieving Neuronal Impairment

Ginseng PDNVs deliver the natural neuroprotective effects of ginseng phytochemicals, potentially alleviating neurodegenerative syndromes, such as stroke and Parkinson's disease, by transferring functional miRNAs to bone marrow stem cells. The upregulation of the PI3K pathway induced neural differentiation and restoration, and promoted the Ras/ERK pathway to enhance nerve regeneration (Xu et al. 2021). Black goji berry PDNVs improved viability and reduced the Bax/Bcl2 ratio in Aβ‐induced PC12 cells by upregulating the PI3K‐AKT pathway (Zhang et al. 2024). Polysaccharide‐rich goji berry PDNVs demonstrated the capacity to prevent muscle strength decline during atrophy by activating the AMPK/SIRT1/PGC1α pathway and myogenic regulatory factors such as MYF5, MYOD, and MYOG in the quadriceps muscle tissue of dexamethasone‐induced atrophy. Alterations in myosin heavy chain isoforms in the quadriceps were reversed through reduced MYH7 and increased MYH4 mRNA expression. Following intramuscular injection into the quadriceps, levels of muscle metabolism regulators and myogenic factors increased, thereby increasing the cross‐sectional area of quadriceps muscle fibers, grip strength, and running distance in dexamethasone‐treated mice (Zhou et al. 2024). Maca PDNVs reversed depression‐like behaviours in model mice, including reducing immobility time, increasing traveling distance, and decreasing eating latency. Neuronal growth was enhanced owing to its ability to modulate TrkB/p‐AKT signalling in the cortex and hippocampus via serum 5‐HT expression. This synergistic effect on enhancing gut microbial diversity and richness was related to an increase in 5‐HT formation (Hong et al. 2023).

### 5.5 Cardiovascular Disorders Therapy

*Taraxacum officinale* PDNVs alleviated chronic intermittent hypoxia‐induced hypertension by enhancing butyrate production, thereby improving gut flora, suppressing systemic inflammatory responses, and inhibiting vascular wall remodelling. Moreover, the number of mucus‐secreting goblet cells and the expression of tight junctions were reduced (Zhang et al. 2023). Cerebral ischemia‐reperfusion, a cardiovascular condition that leads to inflammation and tissue injury in ischemic stroke, was alleviated by functional miR‐156f‐5p‐containing *Panax notoginseng* PDNVs. These nanovesicles entered the brain to reduce cerebral infarct volume, improve behavioural outcomes, and maintain BBB integrity in a tMCAO rat model via M2 polarization and PI3K‐AKT activation. In addition to miRNAs, lipid components exerted antioxidant effects, and saponins were delivered into the brain parenchyma to directly protect the brain (Li et al. 2023).

Bitter melon PDNVs exhibited cardioprotective effects by reducing cardiac injury markers and increasing cardiomyocyte size in doxorubicin‐induced cardiotoxicity after lowering ROS levels, mitigating mitochondrial damage, and decreasing the cardiomyocyte apoptosis rate and p62 ubiquitination through Nrf2 knockdown and p62‐Keap1 inhibition (Ye et al. 2024). Beetroot PDNVs helped prevent heart atrophy and damage by inhibiting ferroptosis through the glutathione cycle. Their anti‐ferroptotic effects were demonstrated by increased antioxidant activity, as evidenced by an elevated GSH/GSSG ratio and higher expression of GPX4 and xCT. Reducing ferroptosis reduces iron accumulation and lipid peroxidation in the heart, thereby lessening systemic damage (Cai and Pan 2024).

### 5.6 Bone Regenerative Effect

Osteoporosis is a systemic skeletal disorder characterized by an imbalance between bone resorption and formation, resulting in bone fragility, a high risk of fractures, trabecular bone loss, and microarchitectural deterioration (Hwang et al. 2023; Zhan et al. 2023). *Pueraria lobata* root PDNVs positively affected osteoblast mineralization and osteoblastic markers in both human bone marrow stem cells and TMAO‐induced mice by modulating autophagy via targeting TMAO in post‐menopausal osteoporosis. Their beneficial phytoestrogenic isoflavones have contributed to the development of potential estrogen replacement therapies (Zhan et al. 2023).

Despite lacking the osteoblast activators of diosgenin and dioscin, oral administration of *D. japonica* PDNVs stimulated osteoblast formation to enhance osteogenesis and prevent osteoporosis by activating osteoblast differentiation and proliferation through the BMP‐2/p‐p38‐dependent Runx2 pathway rather than the ERK1/2 and JNK pathways (Hwang et al. 2023). Ginseng PDNVs, containing ginsenosides Rb1 and Rg1, more effectively inhibited osteoclast differentiation than ginsenosides alone by reducing actin ring formation due to lower toxicity associated with NPV surface lipids. Bone marrow‐derived macrophages were activated via AKT pathway and may inhibit bone cavity formation during inflammatory stimulation (Seo et al. 2023).

### 5.7 Skin Disorders Repairing Effect

*D. morbifera* leaf PDNVs exhibited stronger tyrosinase inhibition than their stem‐derived nanovesicles by reducing MITF, TYR, and TRP‐1 expressions in α‐MSH‐treated B16BL6 melanoma cells. Application to a human skin model further demonstrated superior suppression of melanogenesis and pigmentation compared with arbutin (Lee et al. 2020). Seaweed *Codium fragile* nanovesicles showed stronger inhibition of melanogenesis than *Sargassum fusiforme* nanovesicles in the basal epidermal layer of a 3D artificial skin model without disrupting tissue structure. Tyrosinase, TRP‐1, and MITF expressions were efficiently downregulated by *C. fragile* nanovesicles in MNT‐1 cells, whereas *S. fusiforme* nanovesicles did not downregulate tyrosinase and only slightly reduced TRP‐1 and MITF expression. Both EVs inhibited intracellular tyrosinase activity and reduced α‐MSH‐induced dark spots without affecting MNT‐1 cell viability. Topical application of a prototype cream containing *C. fragile* nanovesicles improved facial skin colour brightness and did not cause skin irritation symptoms (Jang et al. 2021).

Ginsenosides in ginseng PDNVs played a major critical role in inhibiting ROS generation during skin irradiation and oxidative stress. MEK1/2 activity was downregulated by reducing ERK, JNK, and c‐Fos expressions, as well as limiting caspase activation. These actions inactivated AP‐1, thereby reducing MMP activity and limiting skin wrinkling, while simultaneously suppressing inflammation by inhibiting COX‐2 and IL‐6. They showed better inhibition of MMP2 and MMP3 than individual ginsenosides Re, Rg1, Rb1, or Rc (Choi et al. 2024). Skin wound healing is achieved by restoring homeostasis and barrier function, while minimizing inflammatory reactions to reduce infection risk. Ginseng PDNVs promoted skin cell proliferation and cell cycle progression by regulating the ERK and AKT/mTOR pathways and enhancing the expression of cyclins D1 and B1 in HaCaT cells. Furthermore, they modulated proliferative factors (MMP1, fibronectin‐1, elastin‐1, and COL1A1) to accelerate keratinocyte, fibroblast, and endothelial cell growth, while simultaneously inhibiting inflammatory factors (iNOS, COX‐2, and NF‐κB) (Yang, Lu, et al. 2023).

## 6 Biotherapeutic Effect Enhancement of NPVs

Modification of NPVs is an advanced approach for targeted delivery and to achieve superior efficacy (Table 7). Enriching NPVs with specific therapeutic materials through encapsulation can significantly enhance their bioactivity. Structural modification of NPVs, particularly at the membrane surface, can further improve target selectivity and penetration. Innovative applications of advanced technologies on NPVs have been developed to expand their use in various treatments, enhance therapeutic modalities, and improve targeting efficiency. The ability of NPVs as a nanocarrier expands their use for encapsulating therapeutic compounds, while their modifiable lipid bilayer membrane allows for nanovector development and other surface engineering implementations (Li et al. 2024; Shao et al. 2023).

**TABLE 7:** Therapeutic effect of various NPV modifications.

Sources	Modifications	Loaded substances	Disease models	Modification effects	Ref.
Cabbage	Surface modification (HA conjugate)	—	Inflammatory bowel disease	Enhanced intestinal epithelial targeting by binding hyaluronic acid with the CD44 receptor.	Kang et al. (2024)
Ginger	Nanovector (co‐fabricated with FA)	Doxorubicin	Colon cancer	Enhanced anti‐tumor effect by actively targeting folic acid ligands.	Zhang et al. (2016)
Ginger	Biomimetic nanocomposite	Infliximab	GI inflammation	Improved oral efficacy by increasing stability in the GI tract, colon delivery, and intestinal epithelial permeability.	Mao et al. (2021)
Ginger	Encapsulation in poloxamer gel	—	UVA‐induced skin damage	Prolonged topical effect via release rate reduction and extensive skin contact.	Wang et al. (2024)
Grapefruit	Nanovector (co‐fabricated with FA)	Paclitaxel, JSI‐124	Colon cancer	Enhanced permeation for improving accumulation in tumours and prolonged circulation period.	Wang et al. (2013)
Grapefruit	Nanovector (co‐fabricated with FA)	miR‐17	Brain tumor	Improved rapid delivery into the brain and its selective uptake.	Zhuang et al. (2016)
Grapefruit	Fusion nanovesicle (CCR6+ nanovesicle)	CX5461	Glioma	Improved drug loading capacity compared to classical encapsulation and enhanced blood‐brain tumor barrier crossing.	Huang et al. (2023)
*Lycium barbarum*	Encapsulation in a 3D hydrogel implant	Isoliquiritigenin	Spinal cord injury	Promoted motor function recovery and reduced glial scarring for nerve regeneration.	Wang et al. (2024)
*Momordica charantia*	Drug encapsulation	5‐Fluorouracil	Oral carcinoma	Improved therapeutic efficacy and reduced drug resistance.	Yang et al. (2021)
Orange	Surface modification (heparin‐cRGD)	Doxorubicin	Ovarian cancer	Increased transcytosis for enhanced penetration into tumor tissues.	Long et al. (2023)
Spinach	Encapsulation in the chondrocyte membrane	—	Osteoarthritis	Bypassed the endocytic pathway to penetrate cartilage.	Chen et al. (2022)
Watermelon	Encapsulation in PAMAM‐HEXPO complex	miR‐146a	Ovarian cancer	High miRNA loading efficiency and triggered cancer cell membrane leakage via negatively charged nanopores.	Corvigno et al. (2024)

Abbreviations: cRGD, cyclic RGD peptide; FA, folic acid; GI, gastrointestinal; HA, hyaluronic acid; miRNA, micro RNA; PAMAM‐HEXPO, polyamidoamine hybrid, exosomal, polymeric; UVA, ultraviolet A.

### 6.1 Exogenous Substances Encapsulation Into NPVs

EVs have been shown to exhibit biocompatibility, flexibility, and low toxicity in drug delivery, as their lipid‐bound structure can prevent exogenous bioactive molecules from degradation. Their aqueous core can encapsulate hydrophilic drugs, while hydrophobic drugs are enclosed in their lipid bilayer (Lian et al. 2022; Wu et al. 2024). Efficient and convenient encapsulation strategies, either active or passive loading, are crucial for increasing encapsulation efficiency, preserving EV structural integrity, and maintaining the activity of encapsulated molecules during drug delivery (Li et al. 2024; Shao et al. 2023). Passive loading is suitable for lipophilic drugs that pass through vesicle membranes, but it is difficult to encapsulate high‐molecular‐weight substances. Thus, active loading using electroporation, sonication, saponin intervention, or freeze‐thaw cycle can incorporate large biomolecules into vesicles via membrane permeabilization (Rankin‐Turner et al. 2021; Wu et al. 2024). A post‐loading step using ultrafiltration or size‐exclusion chromatography removes unloaded material from EV preparations. Removing unloaded material or drug can avoid biased interpretations of pharmacokinetics and biodistribution during bioactivity evaluation, since the clearance and diffusion of free drug are faster than drug‐loaded EVs. Encapsulation efficiency and loading capacity are influenced by the effectiveness of unloading the drug (Auquiere et al. 2025; Kimiz‐Gebologlu and Oncel 2022).

The encapsulation of 5‐fluorouracil, a chemotherapeutic drug, into bitter melon PDNVs significantly increased the apoptotic rate by 3‐fold and induced more ROS production in oral squamous carcinomas than 5‐fluorouracil alone. The encapsulation method successfully reduced drug resistance during treatment, as NLRP3 and IL‐1β expressions were downregulated. While 5‐fluorouracil alone increased both protein expressions, exhibiting potential drug resistance (Yang et al. 2021). Lipofectamine‐assisted doxorubicin loading into cabbage PDNVs was performed via incubation followed by ultrafiltration to remove unloaded material. This method accelerated encapsulation and improved doxorubicin delivery to the target, thereby suppressing colon cancer growth (You et al. 2021).

Isoliquiritigenin loaded into goji berry PDNVs exhibited a higher encapsulation rate up to 82.6% and a slower half‐release rate than isoliquiritigenin alone at pH 6.8 over 48 h. Its insertion into a 3D‐printed hydrogel spinal cord implant restored normal movement behaviours in mice with spinal cord injury during open‐field experiments by inducing spinal nerve regrowth and reducing tissue edema (Wang, Liu, Cao, et al. 2024). Formulating watermelon PDNVs and polyamidoamine into the HEXPO complex effectively encapsulates miR‐146a, increasing uptake by up to 5.5‐fold and enhancing specific target distribution by 86% in orthotopic ovarian tumor mice. The miR‐146a transfection triggered CD8^+^ T cell immunity in the tumor microenvironment, thereby generating an anti‐angiogenic response (Corvigno et al. 2024).

NPVs are considered an excellent drug delivery system due to their higher stability, lower immunogenicity, higher efficacy, and stronger cellular uptake compared with artificial vesicles. However, they face challenges due to vesicle membrane damage during loading, and their phospholipids limit cargo loading efficiency. Pre‐loading method via transfection and co‐incubation can improve loading efficiency (Bashyal et al. 2022; Li et al. 2024; Moholkar et al. 2023). Standardized loading technique, loading duration, and drug to carrier ratio improve loading efficiency, vesicle integrity, and loaded‐drug bioactivity. Moreover, morphological features, cargo concentration, and loading protocol must be recorded during optimization process (Li et al. 2024; Rankin‐Turner et al. 2021).

### 6.2 Nanovector From NPV Lipids

Nanovectors are nanostructured lipids capable of carrying and delivering bioactive molecules to a target site. Active targeting with nanovectors, which enhances target specificity, is more affective at improving tissue penetration and retention than passive targeting based on natural mechanisms of biodistribution (Dad et al. 2021; Rampado et al. 2019). Nanovectors offer advantages in reducing detectable molecular toxicity to normal cells, targeting specific sites, and improving delivery capacity. Nanovectors are formulated by extracting lipids from EVs using the Bligh and Dyer method, then loading them with bioactive materials via ultrasonication. Sonication enables efficient production steps without the cumbersome techniques required for large‐scale drug loading and nanovector formation (Lian et al. 2022; Wang et al. 2013). However, lipid components in nanovectors may also exhibit bioactivity effects, such as anti‐inflammatory effects, which can interfere with the activity of the loaded drugs and subsequently lead to biased interpretation (Liu et al. 2021).

Applying a polycarbonate liposome extruder membrane reduced the particle size of the doxorubicin‐loaded nanovectors from ginger PDNVs, subsequently increasing the loading efficiency to 95.9% through electrostatic interactions. Hence, it achieved faster doxorubicin release than commercial liposomes at acidic pH, inducing early apoptosis in colon tumours (Zhang, Xiao, et al. 2016). Nanovectors from grapefruit PDNVs also showed potential as a non‐invasive and non‐immunogenic delivery vehicle for miR‐17 into brain tumours within 1.5 h via intranasal administration. The modified vector enhances permeability and retention to improve targeting efficiency (Zhuang et al. 2016). Co‐delivering anti‐cancer drugs with folic acid using nanovectors from grapefruit PDNVs improved stability during uptake by non‐hematopoietic cells. It was highly distributed in the liver and spleen of mice following intraperitoneal and intravenous administration, thereby enhancing tumor growth inhibition with reduced toxicity, as it did not cross the placental barrier in pregnant mice (Wang et al. 2013).

### 6.3 NPV Surface Structural Modification

Manipulation of EV surfaces via a coupling reaction that binds to modified lipid or protein structures is a strategy to enhance efficacy, improve target selectivity, increase biocompatibility, and maintain vesicle membrane integrity (Shao et al. 2023). Consequently, this modification limits the widespread utilization of EVs to accommodate precise targeting. During vesicle surface modification, covalent functionalization of EVs with ligands should be investigated. Combining ligands or polymers with the surface membrane via covalent binding can directly enhance targeting ability and pharmacokinetic profile (Li et al. 2024; Wu et al. 2024).

Heparin‐cyclic peptide complex (cRGD) was inserted into the lipid bilayer membrane of orange PDNVs prior to doxorubicin loading. This modification enhanced doxorubicin release at pH 6.5 and uptake by SKOV3 and 4T‐1 cells, compared to doxorubicin nanoparticles and free doxorubicin. Surface modification also enabled efficient transcytosis, improving tumor tissue penetration and reducing ovarian tumor growth, metastasis, and angiogenesis (Long et al. 2023). The lipid bilayer membrane surface of red cabbage PDNVs was modified by attaching a synthesized hyaluronic acid conjugate, which subsequently lowered the zeta potential from −14.4 to −20.9 mV. Hyaluronic acid demonstrated CD44‐targeting ability, preventing body weight loss and colon length reduction in colitis treatment, without affecting gastrointestinal tract tissues and organs (Kang et al. 2024).

### 6.4 Other Innovative NPV Modifications

Biomimetic nanocomposite modification using inorganic porous structures aims to overcome the disadvantages of EV encapsulation, including low loading capacity, compromised vesicle integrity, and inability to deliver macromolecular drugs. Selecting a nanocomposite framework with a suitable porous structure and size can circumvent limitations on macromolecule loading efficiency and maintain functional integrity. Metal infliximab‐loaded large mesoporous silicon nanoparticles were embedded into ginger PDNVs using ultrasonication. They demonstrated high stability and controlled release of infliximab in the gastrointestinal tract environment, enabling effective oral delivery. NLRP3 inflammasome assembly and activation were inhibited, thereby attenuating body weight loss, colon shortening, pro‐inflammatory cytokine release, and colon damage. Further large‐scale optimization represents a promising oral nano‐herbs delivery platform for alleviating inflammatory bowel disease (Mao et al. 2021; Shao et al. 2023).

A multifaceted modification strategy through combining surface ligand modifications, cargo loading, and membrane fusion can overcome limitations of single modification. However, it is relatively difficult to develop large‐scale applications due to protocol complexity and high‐cost production (Li et al. 2024). Fusion nanovesicles of grapefruit PDNVs were formulated by loading the immunosuppressant CX5461 into grapefruit PDNVs and subsequently combining them with CCR6‐engineered nanovesicles from gingiva‐derived mesenchymal stem cells. This procedure created fusion nanovesicles that alleviated psoriasis and atopic dermatitis through multiple therapeutic mechanisms, including inhibition of ROS generation in macrophages, suppression of keratinocyte inflammation via JAK/STAT downregulation, and prevention of T cells‐CCL20 binding, while promoting Treg cells activity to regulate the immune system (Huang et al. 2023).

Ginger PDNVs were loaded into a poloxamer gel to prolong their topical therapeutic effect by slowing their release rate and maintaining prolonged nanovesicle contact with the skin. The gel acts as a barrier to the gradual release of nanovesicles, preventing possible adverse effects from a single large dose. This modification is suitable for the controlled release of nanovesicles as a topical treatment, which requires meticulous selection of the gel material to achieve excellent biocompatibility and to prevent toxicity from degradation byproducts. Ginger PDNVs‐loaded gel exhibited a better wound closure effect than ginger PDNVs alone on HaCaT keratinocytes and L929 fibroblasts (Wang, Ran, et al. 2024; Zheng et al. 2024). Encapsulation of spinach thylakoid PDNVs into chondrocyte membranes from osteoarthritic cartilage explants allowed penetration into degenerated cartilage, bypassing the endocytic pathway and avoiding lysosomal degradation. This restored intracellular ATP and NADPH levels in chondrocytes. Pairing light irradiation treatment with intra‐articular injection reduced cartilage destruction and attenuated osteoarthritis progression (Chen, Liu, et al. 2022).

## 7 Manufacturing and Biomedical Applications of NPVs

The EV market is currently experiencing exponential growth, driven by a growing understanding of EV roles in therapeutic, diagnostic, and research studies. The global market for EV products in 2021 was valued at $33.1‒57.1 million and is estimated to reach $169.2‒321.9 million by 2026 (Cheng and Kalluri 2023). Growth in EV product value reflects the trend toward precision medicine and the rising popularity of liquid biopsy in the medical field, further boosted by biomedical research investment and advanced technology development (Roy et al. 2018). NPVs are emerging EV‐based therapeutic agents, as they may exhibit minimal toxicity and immunogenicity, high biocompatibility, efficient uptake, and scalable production (Ly et al. 2023). Applying an integrative and sustainable bench‐to‐bedside strategy requires compliance with established regulations to ensure seamless development of NPVs from laboratory research to industrial‐scale manufacturing and clinical translation (Ahn et al. 2022).

### 7.1 Regulatory Compliance

The global regulatory framework for EVs can be categorized into two approaches. The first approach is based on their content for physiological functions, while the second focuses on their source and purification method (Wang, Tsai, et al. 2024). Regulatory units in the USA (US FDA) and Europe (EMA) follow the first approach. The US FDA considers EVs to demonstrate the mechanism of action that regulates therapeutic pathways. Thus, EVs require premarket review for clinical safety and efficacy evaluation, along with chemical manufacturing control. Regulatory units in Japan (PMDA), South Korea (MFDS), and Taiwan (TFDA) follow the second approach, requiring quality control points for raw materials used in EV production (Verma and Arora 2025; Wang, Tsai, et al. 2024). Other regulatory units commonly follow the same regulations as biological medicines or stem cell therapy for EV treatments (Verma and Arora 2025). The uncertainty in regulations can be a barrier for NPV breakthrough, since regulatory approval might be deferred due to different principles and acceptance. Establishing tailored standardized guidelines, including protocols, quality metrics, and assessment methods, in adherence to GMP and unique attributes of NPVs is important for reliable research, manufacturing, and applications (Wang, Zhou, et al. 2024).

MISEV has not provided an established specific guidance section for NPVs since 2014 (Pinedo et al. 2021). Pairing MISEV guidelines with herbal medicinal product regulations may provide a suitable framework for NPV‐based therapeutic products. The multicomponent nature of herbal medicinal products requires detailed characterization using high‐resolution analytical methods to prevent batch‐to‐batch variations and inconsistencies (Lian et al. 2022). Since NPVs represent a relatively new biotherapeutic field, researchers and manufacturers should communicate with regulators to address potential issues and establish comprehensive protocol guidance (Ahn et al. 2022). The research community recommends documenting EV studies on the EV‐TRACK platform to raise awareness of rigor and interpretation of EV parameters and methods. This practice can improve the robustness and integrity of methodology development, supporting protocol standardization and validation (EV‐TRACK Consortium et al. 2017; Wang, Zhou, et al. 2024). Standardization of NPV preparation by following good manufacturing practice (GMP) standards must cover aspects of source selection, isolation, characterization, storage and stability, vesicle loading and modification, administration and targeting, and quality control (Tenchov et al. 2022). Achieving GMP standards, specifications, and facilities before submitting an investigational new drug (IND) ensures safety and quality for the clinical trial (Huang et al. 2025; Zhao, Wang, et al. 2024). In addition, a quality management system (QMS) assures the reliability and reproducibility of standardized procedures by establishing scientific, quality assurance, and quality control activities, such as standard operating procedures, checklists, and review monitoring (Adamo et al. 2021).

### 7.2 NPV Manufacturing

Large‐scale EV manufacturing is a complex process that requires compliance with GMP standards, particularly regarding production parameters, quality control, filling procedures, storage monitoring, and distribution channels (Ahn et al. 2022; Huang et al. 2025). According to the existing standards for EV production, the NPV production workflow can be classified into source selection and pre‐isolation treatment, isolation, and quality control (Chen et al. 2020; Lian et al. 2022). Challenges during mass production of NPVs may arise from source variations, high heterogeneity, inconsistent quality across batches, method scalability, and cargo loading capacity (Tenchov et al. 2022; Wang, Zhou, et al. 2024).

A large amount of natural source material is needed to produce sufficient NPVs for pre‐clinical studies in cells and animals, as well as for clinical trials in humans (Zheng et al. 2025). Despite similar species and phenotype of natural sources, the variability of cultivation procedures, soil or media nutrition, and environmental conditions can affect the physicochemical properties and biomedical effects of NPVs due to potentially different natural components yield and vesicular morphology (Lian et al. 2022). Maintaining standardized cultivation or plantation procedure alongside optimization of quality control must be conducted to ensure uniformity and sustainability of the NPV source (Ly et al. 2023). A smart‐farming model that adopts advanced data‐driven agriculture can be a strategy to standardize NPV sources, improve cultivated yield, reduce metabolite variation, and avoid inconsistent quality (Du et al. 2025). Source biofortification offers an approach to enrich specific high‐concentration components within NPVs to enhance targeted therapy (Wang et al. 2023). Large‐scale plant callus culture and enrichment using a bioreactor may provide a high amount of source (Kimiz‐Gebologlu and Oncel 2022).

The high heterogeneity of NPVs may compromise the morphology, composition, and purity of these nanoparticles. A complex purification procedure yields high purity and low variability in NPVs, but it also makes the manufacturing process more complex and inefficient. An ideal combination of synergistic isolation methods should increase throughput, reduce processing time, and improve yield and purity. Employing a high‐throughput method, such as TFF, improves the scalability of the isolation method by enabling the production of large volumes of concentrated NPV solution and eliminating impurities. A scalable and reproducible isolation method ensures the necessary abundance dose and consistent quality for mass production and clinical translation (Bai et al. 2024). Quantity assessment using reference standards can calculate the amount of encapsulated compounds and determine the purification efficiency and specificity (Wang, Zhou, et al. 2024). Validated sterile practice through disinfecting instruments, aseptic preparation of materials and reagents, controlling particle amount, and aseptic personal technique can diminish microbial contamination to improve biosafety aspect. Optimizing the isolation method should be done in conjunction with quality control assessment to develop a high‐recovery procedure that effectively eliminates impurities and minimizes EV loss during purification (Auquiere et al. 2025).

Enhancing the therapeutic effects of NPVs through cargo and structural modification faces challenges in removing unloaded substances, controlling the amount of loaded cargo, and minimizing vesicle damage. Selecting a modification method based on optimal targeting efficiency and delivering capability may simplify the required modification procedure, minimize the risk of vesicle degradation, and reduce the cost for large‐scale production (Ahmed et al. 2024). Steps of loading or modification, concentration of cargo and reagents, initial particle concentration of NPVs, and carrier solution must be defined and validated to make a reproducible and efficient protocol. Electron microscopy visualization, along with analyses of lipid or protein topography and loaded‐substance concentration, can verify successful modification and post‐loading vesicle integrity. Effective removal of unloaded substances or drugs and the selection of vesicle‐friendly reagents reduce the risk of interference in cargo quantification and misinterpretation of bioactivity results (Rankin‐Turner et al. 2021). Accurate encapsulation efficiency and loading capacity measurements define encapsulation ability of NPVs (Auquiere et al. 2025; Kimiz‐Gebologlu and Oncel 2022).

Optimum temperature, pH, medium, time, methods, and cryoprotectant concentration are critical for maintaining the quality and efficacy of NPVs (Bai et al. 2024). Lyophilization using US FDA‐approved sucrose or trehalose as cryoprotectant is recommended to generate long‐term stability of NPVs (Guarro et al. 2022; Trenkenschuh et al. 2022). A sensitive and robust quality control procedure plays a critical role in identifying batch‐to‐batch variations, supporting scale‐up, and facilitating seamless clinical translation. Standardized and automated manufacturing and quality control may improve throughput, reduce cost, minimize human errors, and enhance reproducibility (Jeppesen et al. 2023; Wang, Zhou, et al. 2024). Criteria of acceptance must be established by considering factors, such as NPV appearance, particle properties, identity markers, metabolites quantity, yield and purity, potency, safety, sterility, and impurities. Comprehensive characterization of bioactive substances inside NPVs is necessary to unravel potential mechanism of action. Validated manufacturing process and comprehensive quality control assessment by respecting GMP regulations and EV preparation guidelines set the foundation for reliable and sustainable clinical translation of NPVs (Ahn et al. 2022; Zheng et al. 2025).

### 7.3 Clinical Trials

Pre‐clinical evaluation uses cells and animal models to study the therapeutic effects and targets of NPVs, as well as their preparation, administration, and delivery methods. It is important to investigate the heterogeneity and modifications of NPVs, as these factors can influence their bioactivity and lead to potential adverse effects. This stage is also critical for assessing the scalability, efficiency, purity, and robustness of isolation and storage protocols to achieve clinical‐scale application standards (Zhao, Wang, et al. 2024). Uptake, biodistribution, targeting efficiency, therapeutic effectiveness, potential immunogenicity and adverse effect, acceptability of dosing and administration route, cargo release, and long‐term safety are crucial factors. These elements are necessary to validate the safety, efficacy, stability, and acceptability of NPVs and to provide predictive data prior to clinical trials (Gupta et al. 2021). Accurate pharmacokinetic interpretations, minimal risk of potential toxicity, and straightforward translations toward humans are enabled by the availability and low species specificity of relevant animal models (Lener et al. 2015).

Rapid drug development relies on clinical trial readiness to design a patient‐centred approach and achieve significant clinical outcomes (LoRusso et al. 2019; Sevagamoorthy et al. 2026). According to the FDA guidance on patient‐focused drug development, clinical trial readiness involves identifying patient priorities, developing fit‐for‐purpose clinical outcome assessments (COA), and addressing trial endpoints (Berg et al. 2024). Clinical trial readiness demonstrated alignment of patient priorities with identified health concepts, concepts of interest (COI), and appropriate COA to support validated protocol development for addressing unmet challenges in clinical trial (Sevagamoorthy et al. 2026). Designing an ideal clinical trial of a drug for a specific disease should recruit a representative number of subjects by considering inclusion and exclusion criteria for the prospective length of the study, using trial endpoints in multi‐site trials equipped with trained personnel (LoRusso et al. 2019).

Following promising pre‐clinical results using cells and animals, NPV developers can apply for an IND before conducting clinical trials. In advance of clinical trials, issues related to quality aspects (characterization, strength, and potency of the material), non‐clinical requirements (in vivo pharmacodynamics, pharmacokinetics, and biosafety), and clinical requirements (clinical trial design and long‐term monitoring) must be resolved through interdisciplinary collaborations (Lener et al. 2015). Therapeutic effectiveness and safety criteria are assessed throughout trial phases, which require elaborate dosing calculations along with pharmacokinetic and pharmacodynamic observations (Cheng and Kalluri 2023). Dose calculations for nanovesicles should consider factors related to the target disease and therapeutic biomarker, route of administration, administered dose and frequency, source and bioactive metabolites quantity, vesicle purity and characteristics, and applied modifications. Establishing uniform dose calculations provides accurate interpretation of bioactivity effects and allows comparison of results between studies, while calibrated quantification instruments ensure dosing accuracy (Gupta et al. 2021; Nguyen et al. 2020).

Nine clinical trials of NPVs have been registered (Table 8) (Chang et al. 2025a, 2025b; Chen et al. 2025; Giancaterino and Boi 2023; Lian et al. 2022; Raimondo et al. 2021). During pre‐clinical studies, oral administration of ginger PDNVs exhibited high biodistribution in the mouse colon to induce anti‐inflammatory effects for reducing acute colitis, enhancing intestinal repair, and preventing chronic colitis and colitis‐associated cancer without toxicity at the local or systemic level (Zhang, Viennois, et al. 2016). The pilot clinical trial of ginger PDNVs and curcumin combination (NCT04879810) aimed to reduce symptoms by at least 30% compared to each single treatment after 28 days of daily oral intake in inflammatory bowel disease patients. However, this study may need more participants to enhance the reliability of its results, as it enrolled fewer subjects than the planned 30 per group. A phase 1 clinical trial of grape PDNVs for delivering curcumin (NCT01294072) to normal and malignant colon tissue is being conducted by administering an oral treatment for 7 days to 15 colon cancer patients and a healthy group. With a relatively short 7‐day observation period, a prolonged safety and tolerability time‐frame study may improve the reliability of the biosafety result.

**TABLE 8:** Registered clinical trials of NPVs.

Trial stage and identifier	NPV therapy	Indications	Interventions	Measured outcomes
Phase 2. NCT07324018.	Oral hydrogel formulated with spirulina EVs.	Radiation‐induced esophagitis.	Oral hydrogel containing spirulina EVs and radiotherapy treatment. Placebo oral hydrogel and radiotherapy treatment.	The incidence of grade ≥2 radiation‐induced esophagitis. Duration and time to onset of grade ≥2 radiation esophagitis. Changes in the composition and alpha diversity of the salivary microbiota. Overall health status and core dimensions of quality of life. Severity of esophageal cancer‐specific symptoms. Adverse events.
Phase 2. NCT07040969.	Oral spray formulated with spirulina EVs.	Radiotherapy‐related oral mucositis.	Oral spray containing spirulina EVs and radiotherapy treatment. Placebo oral spray and radiotherapy treatment.	The incidence, time to onset, and duration of severe (grade ≥3) and all grades of oral mucositis. The longitudinal dynamics of salivary microbiota. Longitudinal analysis of alterations in peripheral blood immune cell profiles. Analysis of inflammatory indicators in peripheral blood and oral saliva. Taste function and xerostomia grade. Oral activities and mouth and throat soreness scores. Overall health status and core dimensions of quality of life. The number of patients who missed 5 or more consecutive radiation fractions. Head and neck cancer‐specific symptoms. Adverse events.
Phase 1. NCT01294072.	Grape PDNVs for curcumin delivery.	Colon cancer.	Dietary supplement of curcumin conjugated with grape PDNVs. Dietary supplement of curcumin. No intervention.	Concentration of curcumin in normal and cancerous tissues. Safety and tolerability of curcumin alone and plant EVs. Metabolic characteristics of normal and tumor colon after treatment. Immune response of colon cancer cells after treatment.
Phase 1. NCT01668849.	Grape PDNVs as an oral therapeutic.	Chemoradiation‐related oral mucositis.	Dietary supplement of grape PDNVs. Drug combination of Lortab, fentanyl patch, and mouthwash.	Extent of pain from oral mucositis. Level of immune biomarkers in blood (cytokines, T cells, NK cells, CD11c, IL12) and mucosal tissue (CD3, CD8, CD11b, F4/80, BrdU).
Pilot study. NCT04879810.	Ginger PDNVs and curcumin combination as oral therapeutics.	Inflammatory bowel disease.	Ginger PDNVs. Curcumin. Ginger PDNVs and curcumin.	Change in inflammation via a reduction in inflammatory cells on colonoscopy. Change in subjective symptom reduction.
Pilot study. NCT04698447.	Lemon PDNVs (CitraVes) as a natural supplement.	Cardiometabolic syndrome.	Natural supplements containing lemon PDNVs for metabolic syndrome subjects. Placebo for metabolic syndrome subjects. Natural supplements containing lemon PDNVs for healthy subjects.	Changes of plasma lipids, waist circumference, BMI, glycemia, and glycated haemoglobin in metabolic syndrome and healthy subjects. Changes of subclinical atherosclerosis and plasma cytokines in metabolic syndrome subjects.
Pilot study. NCT06985121.	Base formulation containing *Centella asiatica* EVs.	Scalp care and hair growth.	Base formula (contains caffeine and panthenol). Base formula plus *Centella asiatica* EVs. Base formula plus IGF‐1 and FGF‐7. Base formula plus *Centella asiatica* EVs, IGF‐1, and FGF‐7. Placebo (without caffeine and panthenol).	Sebum content and scalp condition. Hair length, density, thickness, and loss.
Pilot study. NCT06850935.	Water‐based serum containing *Centella asiatica* EVs.	Skin quality enhancement.	Water‐based serum formulation containing *Centella asiatica* EVs. Water‐based serum formulation without *Centella asiatica* EVs.	Changes in skin hydration, elasticity, and melanin content. Changes in skin wrinkles, skin redness, and skin pore percentages.
Preliminary study. NCT03493984.	Ginger and Aloe PDNVs.	Chronic inflammation in polycystic ovary syndrome.	Ginger PDNVs. Aloe PDNVs. Ginger and Aloe PDNVs. Placebo.	Changes in fasting serum glucose and serum insulin levels during the glucose tolerance test. Changes in serum testosterone and sex hormone‐binding globulin. Level of inflammation markers (CD4, CD8, Foxp3, CD11β, CD33, F4/80, IL‐10, IL‐1β, TNF‐α, IL‐6).

Abbreviations: BMI, body mass index; BrdU, bromodeoxyuridine; CD, cluster of differentiation; FGF‐7, fibroblast growth factor‐7; Foxp3, forkhead box p3; IGF‐1, insulin growth factor 1; IL, interleukin; NK cells, natural killer cells; TNF‐α, tumor necrosis factor‐alpha.

The ability of grape PDNVs to reduce chemoradiation‐associated oral mucositis in head and neck cancer patients was investigated during a phase 1 clinical trial (NCT01668849) by selectively excluding subjects who had previously received chemoradiation therapy to avoid misinterpretation of oral mucositis pain level. Preliminary results from an open‐label pilot study of CitraVes (NCT04698447), a plant‐based product containing lemon PDNVs, demonstrated reductions in LDL and cholesterol levels after 12 weeks of oral administration in subjects with metabolic syndrome (Giancaterino and Boi 2023; Raimondo et al. 2021). To build on this promising evidence, a prospective study with more participants and over a longer duration is necessary to better understand the pharmacokinetic profile and potential adverse effects.

The clinical trial on NPVs faced credibility issues due to limited subject numbers, short observation periods, inadequate trial quality, unmet endpoints, the effect of co‐therapeutic treatment, and personnel unpreparedness. Therefore, a well‐designed clinical trial protocol should follow guidelines, include pre‐clinical evidence, calculate appropriate dosages, assess potential adverse effects, ensure clinical trial readiness, determine a suitable subject number and observation durations, establish clear inclusion and exclusion criteria, define endpoint targets and acceptable parameters, account for co‐therapeutic treatments or drugs, and organize personnel effectively. Standardizing formulations, enhancing regulatory compliance, conducting thorough clinical studies, and practicing ethical advertising are essential for strengthening scientific credibility and ensuring consumer safety.

## 8 Conclusions

The recent increase in publications on natural product‐derived vesicles (NPVs) demonstrates their promising potential as nature‐based nanotherapeutics. Implementing a bench‐to‐bedside research strategy in NPV development is essential for overcoming challenges and achieving targeted outcomes in NPV studies. Source selection, based on prior pharmacological studies, material abundance, and cost‐effectiveness, initiates commendable NPV research. Finding a balance between high‐throughput and less‐destructive techniques provides sufficient isolation material and avoids potential intracellular contamination. Combining complementary isolation techniques provides a highly scalable and reproducible approach for improving the yield, purity, and specificity of NPVs. The use of automated purification methods, together with validated characterization of vesicle morphology, metabolite composition, and specific protein markers, enhances the consistency, selectivity, and reliability of the isolation process. These improvements help minimize inter‐batch variation, reduce vesicle heterogeneity, and decrease variability in clinical outcomes. Furthermore, the identification and selection of protein markers should be guided by species‐specific characteristics and current international recommendations to ensure accurate characterization and standardization of NPVs.

NPVs can encapsulate both natural and exogenous therapeutic substances, delivering them to target sites to alleviate disease and maintain homeostasis. Comprehensive assessment of efficacy, efficiency, and safety in NPVs should consider their stability, cellular uptake, biodistribution, and biosafety during pre‐clinical studies. The observed mechanism of action, dosage, and route of administration can act as fundamental reference points for potential clinical applications. Vesicle modification via exogenous loading or structural alterations offers an innovative strategy to enhance therapeutic efficacy and target selectivity. Despite the lack of specific established regulations for NPV nanotherapeutics, pairing MISEV with herbal medicinal products and current general EV regulations may provide a suitable regulatory framework. Further establishment of tailored NPV regulations is necessary to avoid inappropriate practices, uncertainty of product quality, and delayed regulatory approval. An integrative data‐driven analysis technique, supported by artificial intelligence throughout isolation, characterization, and bioactivity evaluation, provides reliable optimization data for scalable manufacturing and clinical studies. Further application of sophisticated isolation methods, careful observation of safety and efficacy during clinical trials, standardization of specialized protocols and regulations, and integration of a multidisciplinary approach will support the biomedical application of NPVs as advanced nature‐based nanotherapeutics.

## Author Contributions

**Kartiko Arif Purnomo**: data curation; formal analysis; writing – original draft; visualization. **Tsong‐Long Hwang**: conceptualization; writing – review and editing; resources; supervision.

## Conflicts of Interest

The authors declare no conflicts of interest related to this review.

## Data Availability

Data sharing not applicable to this article as no datasets were generated or analysed during the current study.
